# Basidiomycete fruiting body morphology correlates with the convergent loss of photolyase-related proteins

**DOI:** 10.1016/j.isci.2026.117265

**Published:** 2026-08-19

**Authors:** Máté Virágh, Dorottya Anna Szafián, Árpád Csernetics, Xiao-Bin Liu, Dóra Nyári, András Viczián, László G. Nagy, Zsolt Merényi

**Affiliations:** 1 Synthetic and Systems Biology Unit, Biological Research Centre, Hungarian Research Network (HUN-REN), Temesvári Krt. 62, 6726 Szeged, Hungary; 2 Department of Biotechnology and Microbiology, University of Szeged, Közép fasor 52, 6726 Szeged, Hungary; 3 Institute of Plant Biology, Biological Research Centre, Hungarian Research Network (HUN-REN), Temesvári Krt. 62, 6726 Szeged, Hungary; 4 Korea University, Seoul, Republic of Korea

**Keywords:** cryptochrome, photolyase, photoreactivation, *Coprinopsis cinerea*

## Abstract

Cryptochromes and photolyase-related (CRY/PHR) proteins are flavoproteins involved in blue-light signaling and/or the light-dependent repair of UV-induced DNA damage across all domains of life. Although well-studied in many eukaryotes, their evolution and function in mushroom-forming fungi (Agaricomycetes and Basidiomycota) remain poorly understood. By analyzing 655 genomes, we show that fungi ancestrally possessed four CRY/PHR subfamilies, of which class I cyclobutane pyrimidine dimer (CPD) photolyases became broadly conserved across Dikarya. Class I CPD photolyase-related proteins have been lost in Agaricomycetes with gasteroid fruiting bodies, where sexual spores develop shielded from light. Functional characterization of the class I CPD protein Phr1 of *Coprinopsis cinerea* confirmed its light-dependent photorepair function. We further demonstrated that *phr1* expression is upregulated by blue light in a white-collar-complex-dependent manner. Overall, our results provide an example of parallel gene loss associated with a morphological change and shed new light on the evolution of the CRY/PHR protein family.

## Highlights

• CRY/PHR diversity has been reduced in mushroom-forming fungi
• Loss of class I CPD correlates with the evolution of enclosed fruiting body form
• Phr1 of *Coprinopsis cinerea* contributes to photoreactivation
• Expression of *phr1* is regulated by blue light in a WCC-dependent manner

## Graphical abstract

**Figure:**
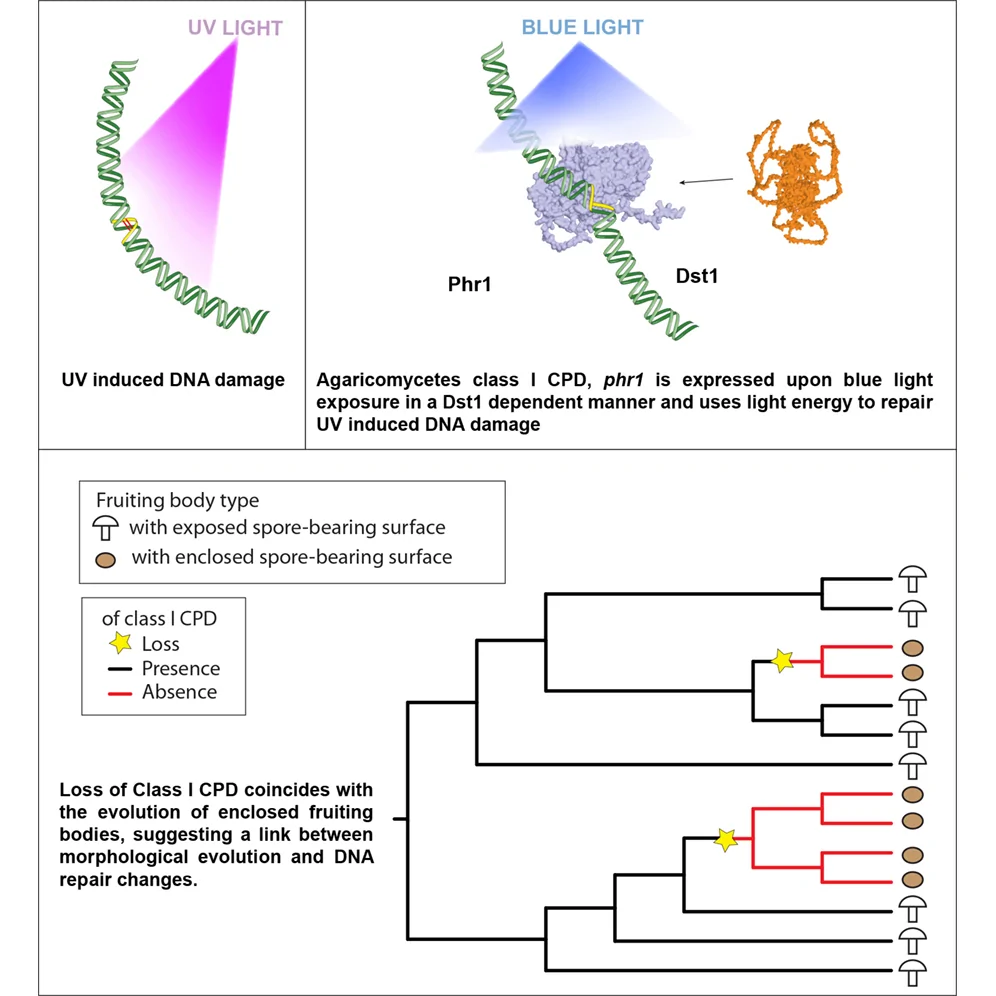

## Teaser

Biological sciences; Molecular biology

## Introduction

Cryptochromes and photolyase-related (CRY/PHR) proteins are widely conserved blue-light-dependent flavoproteins that share common ancestry but possess distinct functions.^1^ While CRY entrain the circadian clock in plants and animals or regulate morphogenesis and development,^2^ photolyases repair UV-induced DNA damage via photoreactivation.^3^ CRY/PHR proteins can be classified into multiple distinct subfamilies^4^ originating from a common ancestor that is suggested to be a class I CPD (cyclobutane pyrimidine dimer) photolyase-like protein, potentially involved in UV-induced DNA damage repair.^5^ These subfamilies show a patchy distribution along the tree of life^4^ and often provide a clear example of how changing selective pressures associated with light-related lifestyle changes can drive expansion,^6^ contraction,^7^ and functional diversification of protein families.^8^^,^^9^

The fungal CRY/PHR repertoire was reported to be restricted to four subfamilies (class I CPDs, (6-4)-PHRs, CRY-DASH, and class II CPDs in the Microsporidia clade).^4^^,^^6^ Members of these subfamilies are studied mostly in Ascomycota and are often regulated by the widely conserved blue-light photosensor, the white-collar complex (WCC),^10^^,^^11^ and possess diverse functions, such as light-dependent DNA repair^10^^,^^11^^,^^12^^,^^13^^,^^14^ and regulation of diverse processes, such as the sexual and the asexual cycles.^15^^,^^16^^,^^17^ Less is known about the CRY/PHR repertoire of Basidiomycota, including mushroom-forming fungi (Agaricomycetes).

Mushrooms, or fruiting bodies, are the sexual reproductive structures of Agaricomycetes in which the spore-bearing tissue (hymenium) may be located either on the surface (as in resupinate and coralloid/clavarioid forms) or in highly organized downward-oriented structures such as the pores and lamellae (trama) of the pileate-stipitate and pileate-sessile forms (see morphologies, e.g., in Virágh et al.^18^). In most cases, these structures are exposed to light and other environmental factors. Certain Agaricomycetes species develop spores in completely closed fruiting bodies, protected by a peridium that shields the developing spores.^19^ These include so-called gasteroid and secotioid species, which can also be divided into epigeous—such as puffballs, stinkhorns, and bird’s nest fungi—and hypogeous species (e.g., false truffles), based on whether their fruiting bodies develop above or below ground, respectively. The “secotioid syndrome” hypothesis, formulated by Henry Thiers,^20^ posits that gasteroid species evolved repeatedly several times from pileate-stipitate forms, suggesting an adaptive value of gasteromycetation (i.e., the transition of a fruiting body from pileate-stipitate to a gasteroid). Indeed, recent evidence suggests that gasteroid forms evolved more than 100 times within Agaricomycetes.^21^ Yet, the genetic changes that may correlate with these morphological transitions remain poorly understood.^22^^,^^23^^,^^24^

Here, we explore the diversity and function of CRY/PHR proteins based on a large fungal dataset, with a special focus on mushroom-forming Agaricomycetes. Using comparative genomics, we reveal that CRY/PHR proteins show a patchy distribution among fungi and that most mushroom-forming fungi harbor a single member of the family that is a class I CPD protein. Furthermore, we demonstrate that most gasteroid species have convergently lost this single class I CPD protein, which may be related to their enclosed spore production. Using reverse genetics, we also show that the class I CPD protein Phr1 of *C. cinerea* contributes to photoreactivation and is regulated by blue light via the blue-light sensor WCC. This indicates that Phr1 of *C. cinerea* retained its ancestral photolyase function, and it becomes dispensable in gasteroid species. Our results provide an example of a parallel genetic change accompanying the convergent emergence of gasteroid fruiting bodies and give a novel example of lifestyle/morphology-related changes in the CRY/PHR family.

## Results

### Class I CPD is the most prevalent CRY/PHR protein in mushroom-forming fungi

**Figure 1:**
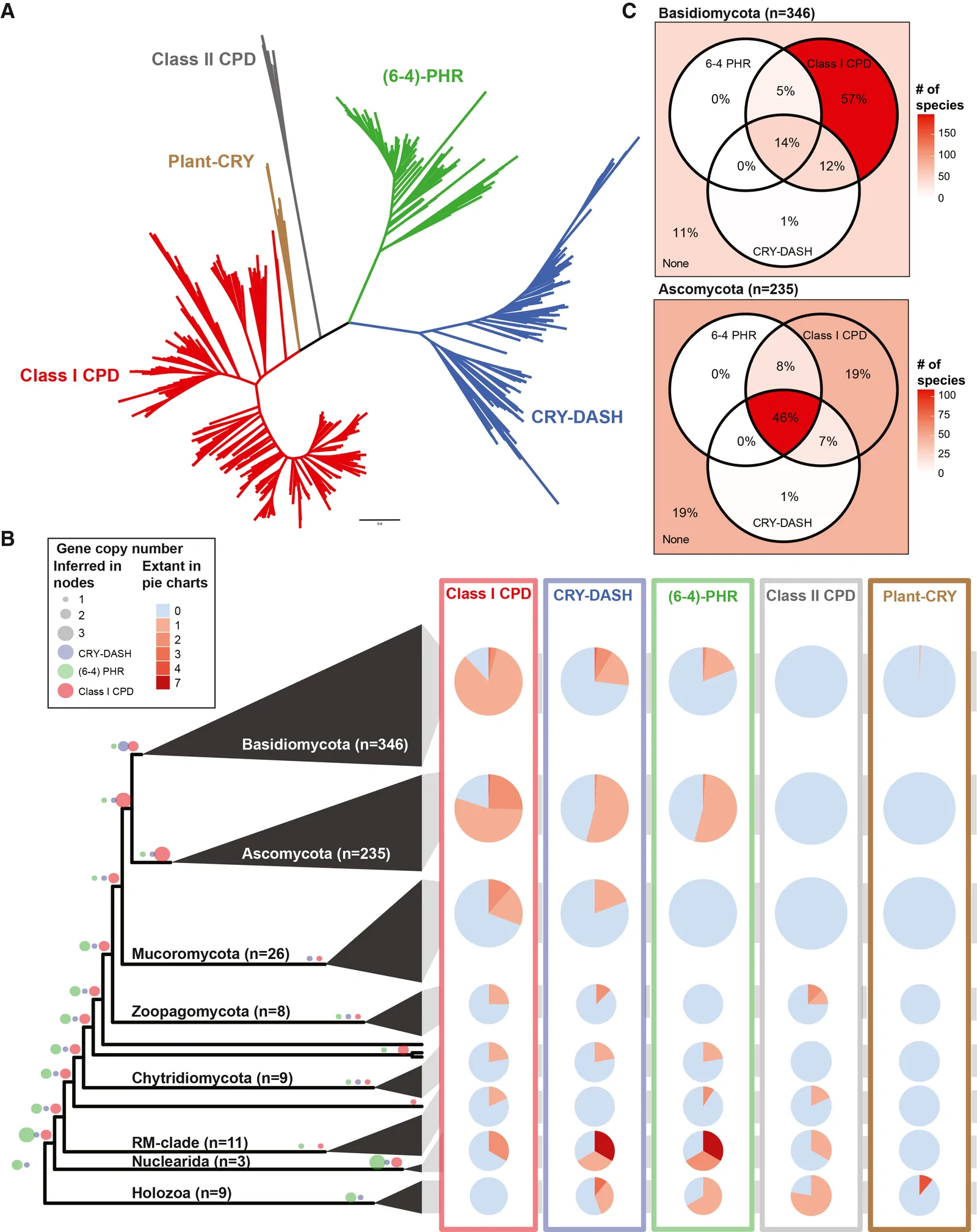
Distribution of CRY/PHR cryptochromes in fungal genomes (A) Gene tree of the CRY/PHR family colored by the identified subfamilies. For a detailed gene tree, see Figures S1 and S2. The scale bars indicate the number of amino acid substitutions per site. (B) Proportions of species in Ascomycota and Basidiomycota containing different combinations of the three most abundant CRY/PHR subfamilies. The white-to-red gradient indicates the raw number of genomes associated with each subfamily combination represented in the Venn diagram sections. (C) Distribution of cryptochrome subfamilies across the fungal tree of life. The left side of the figure shows a simplified species phylogeny for our 655-genome dataset. Colored bubbles at internal nodes represent the inferred ancestral copy numbers of the three most abundant CRY/PHR subfamilies: class I CPD (red), CRY-DASH (blue), and (6-4)-PHR (green). Internal nodes were not visualized for class II CPD (gray) and plant CRY (brown). Pie charts indicate the relative proportions of recent copy numbers of these subfamilies belonging to each collapsed taxonomic group.

To obtain a comprehensive overview of the CRY/PHR family, we performed homology searches using a defined set of 499 CRY/PHR reference sequences^4^ against a dataset of 655 opisthokont genomes. In total, we identified 1,131 genes belonging to the CRY/PHR family (Tables S1 and S2). To resolve the evolutionary relationships of these genes, we inferred gene phylogenies for the complete CRY/PHR family, in which we identified five out of the seven eukaryotic CRY/PHR subfamilies that we used for reference. In fungi, we detected CRY-DASH, (6-4)-PHR, class I and class II CPDs, and plant CRY (Figures 1A, S1 and S2 and Table S2).

To investigate the evolutionary history of CRY/PHR genes in fungi in more detail, we reconstructed the duplication and loss histories of each subfamily separately. Using Dollo parsimony, we inferred ancestral gene copy numbers that we mapped onto a species phylogeny of the 655-genome dataset (Figures 1B, S3 and S4). These analyses indicate that the last common ancestor of fungi possessed CRY/PHR genes from four subfamilies: CRY-DASH, (6-4)-PHR, and class I and II CPD. In contrast, the plant CRY subfamily was likely acquired by fungi via horizontal gene transfer (see below).

Although CRY-DASH, (6-4)-PHR, and class I CPD are found in nearly all extant fungal phyla, their prevalence within individual phyla is generally low (< 50%; Figure 1B). This patchy distribution can be explained by extensive lineage-specific gene losses within each subfamily (Figure S3). The fourth subfamily, class II CPD, appears to have been completely lost before the divergence of Mucoromycota, Ascomycota, and Basidiomycota (Figure 1B).

Class I CPD remains the most consistently retained subfamily across fungi. This pattern is particularly pronounced in Ascomycota and Basidiomycota—the phyla containing most fruiting-body-forming species—where class I CPD is present in 80.0% and 88.2% of species analyzed, respectively. In Ascomycota, 61% of species retain at least one additional subfamily (CRY-DASH or (6-4)-PHR), whereas in Basidiomycota, and especially in Agaricomycetes, 57% and 71% of species possess only class I CPD proteins, respectively (Figure 1C and Table S1). These patterns suggest the reduction of CRY/PHR diversity in mushroom-forming fungi (Agaricomycetes).

In addition to the aforementioned fungal CRY/PHR repertoire, we identified plant CRY homologs in a single fungal lineage: *Malassezia* (Basidiomycota). To confirm their phylogenetic placement, we reconstructed a separate gene tree including fungal, eukaryotic, and prokaryotic plant CRY sequences. In this analysis, *Malassezia* plant CRYs clustered within prokaryotic lineages with high support (closest match: *Kocuria* sp.), suggesting the possibility of horizontal gene transfer from Gram-positive bacteria (Figure S5).

### Class I CPD occurrence correlates with fruiting body morphology in mushroom-forming fungi

Given the predominant retention of class I CPD in mushroom-forming Agaricomycetes, subsequent analyses focus specifically on this subfamily in Agaricomycetes. Of the 270 analyzed Agaricomycetes genomes, 90.0% (243 genomes) possess a class I CPD gene, of which 87.4% (236 genomes) contain exactly one copy, indicating that this subfamily is conserved and generally single-copy (Figure S4). The seven species with two or three copies are scattered across the Basidiomycota phylogeny, reflecting species-specific duplication events. Consistent with this, the inferred ancestral copy number of class I CPD was one in most internal nodes.

**Figure 2:**
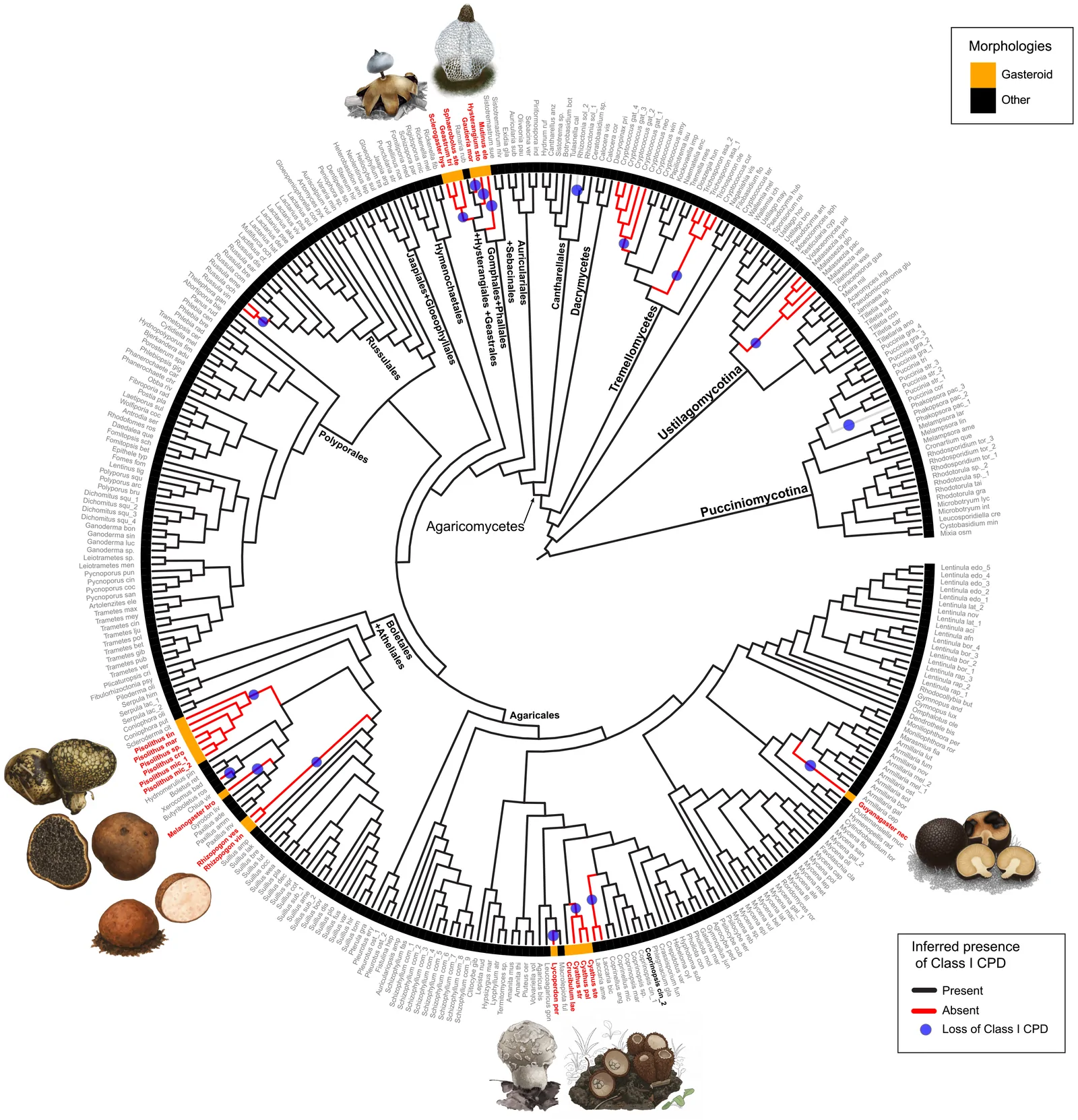
Convergent loss of class I CPD in species with gasteroid fruiting bodies Species phylogeny of Basidiomycota showing the inferred evolution of the class I CPD subfamily. Class I CPDs have undergone losses (blue circles), most frequently in lineages with gasteroid fruiting body morphologies (orange part of the outer ring), where spores develop inside enclosed fruiting structures. Apart from gasteroid lineages, we detected four losses of class I CPD in species with no or pileate-stipitate morphology. Branches inferred to contain class I CPD are shown in black, and branches lacking it are shown in red. *Coprinopsis* cin_2 in bold represents the species used for functional experiments. Drawings of *Lycoperdon* sp., *Geastrum* sp.*,* and *Phallus* sp. were modified from Nagy,^25^ while drawings of *Guyanagaster* sp.*, Cyathus* sp.*, Rhizopogon* sp.*, Melanogaster* sp.*,* and *Pisolithus* sp. were generated with the assistance of ChatGPT (OpenAI).

Despite this general conservation of class I CPDs in Agaricomycetes, 27 species (10.0% of all Agaricomycetes) lack class I CPDs (Figures 2 and S6 and Table S1). Notably, 21 of these (80.8%) have gasteroid morphology. Our dataset includes 10 independent lineages with gasteroid morphology, three from Agaricales (*Guyanagaster*, *Lycoperdon*, and *Cyathus* species), three from Boletales (*Rhizopogon*, *Melanogaster*, *Pisolithus*, and *Scleroderma* species), and one from each of the following orders: Phallales (*Mutinus* sp.), Geastrales (*Sclerogaster*, *Geastrum*, and *Sphaerobolus* species), Gomphales (*Gautieria* sp.), and Hysterangiales (*Hysterangium* sp.). A shared feature of these diverse lineages is that the spore-bearing tissue develops within the fruiting body, shielded from direct light. Regardless of the specific morphological type, all ten clades show a loss of class I CPD. The only gasteroid species in which we found a class I CPD gene is *Crucibulum laeve* (common bird’s nest), but its sister species (*Cyathus* spp.) have lost it. Consequently, 95.5% of the studied gasteroid species have lost their class I CPD.

In contrast, the absence of class I CPD is rare in other Basidiomycota morphologies. Among non-gasteroid species lacking class I CPD, we identified a *Rhizoctonia* species (which has no fruiting bodies) and five pileate-stipitate species (two *Laccaria*, two *Russula*, and one *Butyriboletus*), representing only 3.4% of the 145 studied pileate-stipitate taxa. All species with resupinate, coralloid, clavarioid, and pileate-sessile morphologies (altogether 98 studied species) possess class I CPD. This suggests that loss of class I CPD is specifically enriched in gasteroid lineages.

This pattern appears specific to gasteroid Basidiomycota. In gasteroid Ascomycota, class I CPD was retained in four out of five examined species, and all three gasteroid Mucoromycota species possess class I CPD (Table S1). Thus, while gasteroid morphology is strongly associated with class I CPD loss in Basidiomycota, this trend is not observed in other fungal phyla, indicating that additional, lineage-specific factors likely underlie this pattern.

To assess whether the absence of class I CPD in certain Basidiomycota morphologies reflects correlated evolution, we performed a phylogeny-aware analysis, encoding both the presence/absence of class I CPD and fruiting body morphology as binary traits. A strong signal of correlated evolution was observed for gasteroid fruiting body forms (likelihood ratio = 32.4, *p* = 1.58 × 10^−6^). We, therefore, hypothesize that the function of class I CPD in Basidiomycetes may be linked to processes dispensable in species with gasteroid fruiting bodies.

Finally, to ensure that the parallel loss of class I CPD is not merely a result of a potential massive gene loss in gasteroid lineages, we searched for genes that are highly conserved among Agaricomycetes (>75% prevalence) but missing from at least 25% of gasteroid species (Table S3). This analysis identified only 12 genes, including the class I CPD. This suggests that only a few genes are consistently lost in parallel across these lineages. Together, these results provide, to our knowledge, the first evidence of molecular correlates of the evolution of gasteroid fruiting bodies.

### Functional interrogation of the class I CPD protein in *C. cinerea*

To home in on the exact function of class I CPD protein in Agaricomycetes, we disrupted the class I CPD protein-encoding gene, *phr1*, in the mushroom-forming model fungus, *C. cinerea* (JGI Mycocosm protein: 492962; from here, the disruptant strain is referred to as *Δphr1*). To this end, we used a homology repair template (plasmid: *Ccphr1*KO) and an in vitro-assembled RNP-based CRISPR-Cas9 system.^26^ Out of 48 transformants, we selected, isolated, and validated two independent disruptants by PCR and Sanger sequencing, confirming the insertion of the wild-type (WT) *C. cinerea para*-aminobenzoic acid synthase gene, which was used as a selection marker. (Figures S7A and S7B). We additionally generated and selected two overexpression lines (referred to as *phr1*OE) for downstream analysis with an ectopically integrated *phr1* overexpression cassette (plasmid: *Ccphr1*OE), where the *phr1* gene is driven by the constitutive *Agaricus bisporus gpdII* promoter in a WT background. The overexpression resulted in a median 105-fold (interquartile range [IQR]: 92–265, *p* value = 0.0160, one-sample *t* test) and a median 35-fold (IQR: 28–72, *p* value = 0.0103, one-sample *t* test) increase in relative expression level in the dark, compared to the WT strain (Figure S7C) and smaller or no expression level changes under the blue light (median: 1.1, IQR: 0.9–1.8, *p* value = 0.250; and median: 1.6, IQR: 1.4–1.7, *p* value = 0.07, one-sample *t* test; Figure S7C), which we explain by the high original expression level of *phr1* in the WT under these circumstances. We selected one overexpression strain for subsequent developmental observations and relative expression measurements.

### Phr1 shows photolyase activity *in vivo* in *C. cinerea*

Fungal class I CPD proteins generally function as *bona fide* photolyases.^10^^,^^27^ Previously, it was shown that a 1-h-long light treatment following UV exposure significantly increases the survival rates of the vegetative spores (oidia) of *C. cinerea,* showing that photoreactivation exists in this species.^28^ To test whether *C. cinerea* Phr1 mediates photoreactivation, we measured the survival rates of oidia in both Δ*phr1* and WT strains following UV-B exposure, with and without subsequent light treatment.

**Figure 3:**
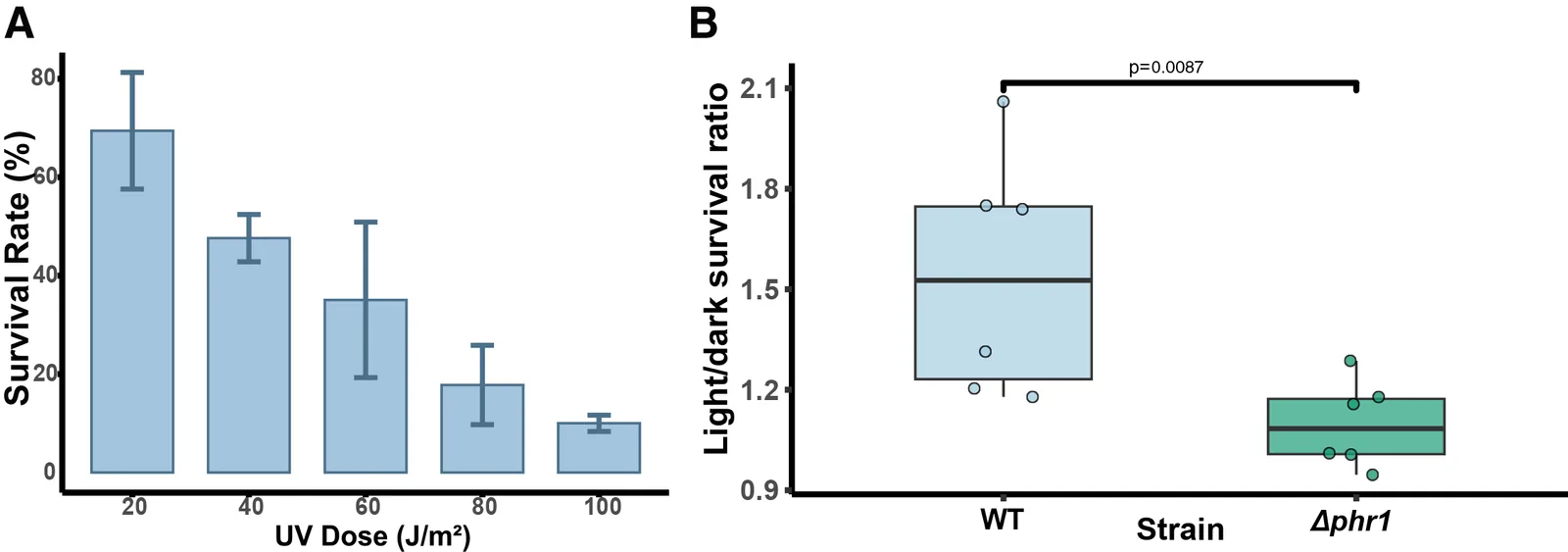
Phr1 contributes to photoreactivation in *C. cinerea in vivo* (A) Susceptibility of *C. cinerea* oidia (asexual spores) to different UV doses was assessed by determining their survival rate after UV treatment of different doses. To this end, approximately 500 oidia were spread on YMG agar plates and moved to the dark at 28°C for 48 h. Colony-forming units (CFUs) were counted and compared with the number of CFUs formed by the untreated control, with the latter treated as 100%. Each bar represents the mean survival rates of spores following treatment with different UV doses, calculated from three independent biological measurements (*n* = 3), each with five technical replicates (k = 5). (B) To assess the role of *C. cinerea* Phr1 in photoreactivation, we determined the light/dark survival ratio of both the WT and the Δ*phr1* strain after UV exposure. Approximately 500 oidia were plated onto YMG agar medium and subjected to a UV dose of 60 J/m^2^. After irradiation, plates were either subjected to 1 h of light treatment before incubation in the dark at 28°C for 48 h or moved immediately to the dark. The light/dark survival ratio was determined for the wild-type and Δ*phr1* strains by dividing the survival rate of UV-exposed spores after light treatment by the survival rate without light treatment. Each point on the IQR box plots represents the light/dark survival ratio from one of six biological replicates (*n* = 6), with each biological replicate calculated as the mean of five technical replicates (k = 5). Statistical significance was assessed using a Wilcoxon signed-rank test.

First, to assess UV susceptibility in the WT, we exposed WT oidia to a range of UV doses (0–100 J/m^2^) and calculated survival rates by comparing the number of colony-forming units (CFUs) of the UV-treated samples to the untreated controls (Figure 3A). We selected the dose of 60 J/m^2^ for further subsequent experiments. We then tested the effect of a 1-h-long light treatment on the survival rates of spores of the Δ*phr1* and the WT strains. To this end, we calculated the light/dark survival ratio of the WT and the disruptant strains by dividing the survival rates of the UV-exposed and subsequently light-treated oidia by the UV-exposed samples kept in the dark (Figures 3B and S8). Light/dark ratios greater than one indicate photoreactivation. The WT strain showed an average light/dark survival ratio of 1.54 (±0.36, *n* = 6, k = 5), showing that photoreactivation occurred in the WT, while the mean light/dark survival ratios of the two independent disruptants were 1.09 (±0.13, *n* = 6, k = 5, Figure 3B) and 0.93 (±0.14, *n* = 6, k = 5, Figure S8), showing that light treatment did not affect the survival rate of spores of the disruptants after UV exposure in the mutants. The significant differences (*p* = 0.0087 and 0.0022, Wilcoxon signed-rank test) in the light/dark survival ratios between the WT and the disruptant strains indicate that Phr1 of *C. cinerea* contributes to photoreactivation.

### The expression of *phr1* is blue light and Dst1-dependent

The expression of *phr1* of *C. cinerea* is upregulated upon light induction (Figure S9).^29^ The WCC is known to be involved in numerous light-related processes^30^^,^^31^ and regulates the expression of CRY/PHR genes in several fungi.^10^^,^^11^^,^^32^ Based on these clues, we aimed to measure the relative expression of *phr1* under different light conditions and its dependence on the WCC.

**Figure 4:**
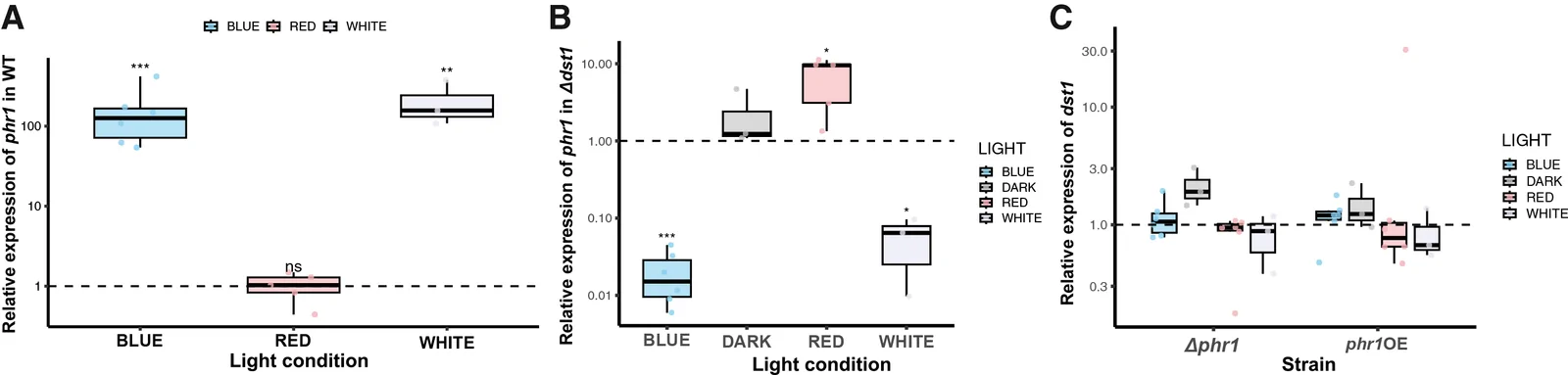
Transcription of *phr1* is induced differentially under different wavelengths of light in a Dst1-dependent manner (A) Expression of *phr1* in WT *C. cinerea* was determined after 2 h of exposure to monochromatic blue (λmax = 470 nm, 40 μmol m^−2^ s^−1^), monochromatic red (λmax = 667 nm, 40 μmol m^−2^ s^−1^), and white light (25μmol m^−2^ s^−1^), relative to its expression level in constant darkness. (B) Expression of *phr1* was determined in the Δ*dst1* strain after the same irradiation as described in (A). The relative expression level of *phr1* in the Δ*dst1* strain was determined relative to the *phr1* level in the wild-type (WT) strain under the respective irradiation in the WT. (C) Relative expression of *dst1* in the Δ*phr1* and in the *phr1*OE strain was determined relative to the expression of *dst1* in the WT strain under the same irradiation as described in (B). The expression of *dst1* is normalized to the *dst1* level, determined under the respective irradiation in the WT. Individual dots on the IQR boxes represent the means of biological replicates (*n* = 3–5) calculated from two technical replicates in each batch (k = 2). ∗*p* < 0.05, ∗∗*p* < 0.005, ∗∗∗*p* < 0.0005, one-sample *t* test.

First, we assessed *phr1* expression in the WT strain after 2 h of exposure to monochromatic blue and red light, under white light, and in the dark, using real-time qPCR (Figures 4A and S10). Compared to darkness, blue and white light significantly increased *phr1* expression (74.23-fold, *p* = 0.00487, and 138.96-fold, *p* = 0.00594, respectively), while red light had no significant effect (Figure 4A).

Next, we investigated how Dst1, the *C. cinerea* ortholog of the photoreceptor component Wc1 of the WCC complex (JGI Mycocosm protein: 369621), affects *phr1* expression*.* To this end, relative expression levels of *phr1* in the *Δdst1* background in the dark, under monochromatic blue and red light, and under white light were compared to those in the WT under the same light conditions (Figure 4B). We found that the relative expression levels of *phr1* under blue and white light (fold changes: 40.7 and 17.86, *p* values = 0.00006 and 0.0004, respectively; one-sample *t* test) were significantly lower in the *Δdst1* strain compared to the WT, but did not change in the dark and was significantly higher (fold change: 9.08, *p* = 0.00041, one-sample *t* test) upon red light illumination. This shows that *phr1* expression is regulated by blue light in a Dst1-dependent manner and that Dst1 might contribute directly or indirectly to the repression of *phr1* in red light.

Some fungal CRY/PHR proteins are also known to inhibit the expression of WCC.^33^ To find out whether Phr1 regulates *dst1* expression in *C. cinerea*, we measured the relative expression level of *dst1* in the Δ*phr1* background and in the *phr1* overexpression strain *phr1OE* under various light conditions and compared them to that of the WT. The expression of *dst1* was not altered either in the disruptant or in the overexpression strain under any applied light conditions (Figures 4C and S10). This shows that, unlike in many fungi, Phr1 does not contribute to the regulation of *dst1* expression in *C. cinerea*.

### Phr1 has no obvious effect on developmental processes in *C. cinerea*

Though most fungal class I CPD proteins act as photolyases, in some species, they have gained regulatory roles in photoreception,^8^^,^^17^ stress response,^8^ and development.^15^ To test whether *C. cinerea* Phr1 contributes to the regulation of development, we tested the effect of the disruption and the overexpression of *phr1* on vegetative growth (Figure S11), oidia production (Figure S12) under different light conditions, and fruiting body formation. Neither disruption nor overexpression has an obvious effect on any of the aforementioned developmental processes in *C. cinerea.*

## Discussion

Members of the CRY/PHR protein family are descendants of ancestral flavoproteins that repair UV-induced DNA damage, play a role in photoreception, and entrain the circadian clock in different species.^2^ Studies with a broader focus on eukaryotes showed that most fungi harbor members of three subfamilies of the CRY/PHR family: class I CPD, (6-4)-PHR, and CRY-DASH, while the occurrence of class II CPD is limited to the Rozellida-Microsporidia clade.^4^^,^^6^ Overall, our fungal-specific dataset refines this view by revealing both novel (plant CRY) and extended subfamily occurrences (class II CPD) and by resolving their patchy distribution (especially in CRY-DASH and (6-4)-PHR subfamilies). These findings also confirm class I CPD as the most conserved CRY/PHR subfamily in fungi, in contrast to other eukaryotic lineages where they are mostly absent.^4^^,^^6^

Conservation of class I CPD is strongest in Ascomycota and Basidiomycota, the two phyla that include the majority of fruiting-body-forming species. In Ascomycota, the CRY/PHR repertoire remained diverse, with substantial representation of (6-4)-PHR and CRY-DASH proteins alongside class I CPDs. In contrast, we show that in the Basidiomycota, extensive losses of (6-4)-PHR and CRY-DASH proteins (43 and 36 losses, respectively; Figures S3, S4, and S6) have reduced CRY/PHR diversity, especially in Agaricomycetes. Coinciding with this reduction, class I CPDs are present in 90.0% of species (with 14 losses) and often represent the sole remaining member of the CRY/PHR family in the Agaricomycetes (Figures 2 and S6). Therefore, we concluded that in most mushroom-forming species, class I CPD represents the only remaining CRY/PHR with the potential to fulfill CRY and/or photolyase functions.

Strikingly, we found a significant correlation between the loss of class I CPD and the evolution of gasteroid fruiting body forms within Agaricomycetes. Although class I CPDs are highly conserved across Agaricomycetes with diverse fruiting body morphologies, they showed losses in all ten independently evolved gasteroid lineages and are absent from 95.5% of the gasteroid Basidiomycota species analyzed here (Figure 2).

Gasteroid fruiting bodies evolved multiple times independently within Agaricomycetes and are thought to represent adaptations to environmental conditions that favored the enclosure of the spore-bearing surface into an enclosed fruiting body.^20^ These forms are generally considered evolutionarily terminal states, with reversals being exceedingly rare,^18^^,^^21^ potentially due to the loss of complex traits such as forcible spore discharge and highly organized tramal architecture.^18^ However, the extent of parallel gene loss among gasteroid lineages is low (Table S3). Therefore, the convergent absence of class I CPD across independently evolved gasteroid taxa is unlikely to represent a stochastic consequence of broad gene loss associated with morphological transition. Instead, its convergent loss suggests that the function of class I CPD became consistently dispensable during the evolution of gasteroid fruiting bodies. To clarify which function might become dispensable in gasteroid species, we examined the role of Phr1 in a non-gasteroid Agaricomycetes model.

Available developmental transcriptomic data from several mushroom-forming fungi suggest that a potential link between the photolyase function of Phr1 and its loss in gasteroid lineages may be explained by its spatial expression pattern in fruiting bodies (Figure S13).^34^ *Phr1* and its orthologs show an expression peak in the hymenium (Figure S13), suggesting they function in the spore-producing tissues. Gasteroid fungi develop spores entirely within the fruiting body, protected from direct environmental exposure. This raises the possibility that shielding spore production from light may reduce the need for molecular UV-protection mechanisms. Under this scenario, photolyases, such as Phr1, may have become less critical in gasteroid taxa, leading to relaxed selection and ultimately gene loss.

Using reverse genetic approaches in *C. cinerea*, we demonstrated that Phr1 contributes to photoreactivation (Figure 3B) and is expressed upon blue-light induction in a WCC-dependent manner (Figures 4A and 4B).

Most fungal class I CPD proteins act as canonical photolyases.^11^^,^^15^^,^^27^ Within Basidiomycota, the only well-characterized class I CPD protein, the *Ustilago maydis* Phr1, is a *bona fide* CPD photolyase.^10^ This, together with our data, suggests that the Agaricomycetes Phr1 proteins retained their ancestral photolyase function, facilitating photoreactivation in mushroom-forming fungi. Indeed, photoreactivation was reported in at least two mushroom-forming fungi, *C. cinerea*^28^ and *Schizophyllum commune*.^35^ Based on these, we can further speculate that the common ancestors of independently evolved gasteroid species harbored a class I CPD protein with the same function as a photolyase involved in photoreactivation.

Nevertheless, there is some indication that spore protection from light alone may not fully account for the observed evolutionary patterns. First, no comparable pattern in the lack of class I CPD was found in gasteroid morphologies among Ascomycota or Mucoromycota. Second, in Agaricomycetes, partial and universal veils also protect the spore-producing surface during the early stages of fruiting body development. However, class I CPD genes are retained in fungi that produce veils (e.g., *Volvariella* spp., *Amanita* spp.). Other forms of protection against light-induced damage, such as strong pigmentation,^36^^,^^37^ thick fruiting bodies, and preference for shaded habitats,^38^ also do not appear to correlate with class I CPD loss. Although the extent to which UV radiation reaches the hymenium and developing spores in such various fungi remains unclear,^38^ the widespread accumulation of photoreactive and photosensitizing pigments in fruiting bodies and spores suggests that light may still play an important physiological role even within these seemingly protected microenvironments.

Aside from UV protection, class I CPD may have alternative roles that could have become dispensable in gasteroid fungi. Two recent studies suggested the involvement of CPD/PHR proteins in the protection against oxidative DNA stress.^8^^,^^39^ The CPD/PHR protein of the blind cavefish *Phreatichthys andruzzii* repairs CPD lesions as well as 8-hydroxydeoxyguanosine damage independently from light.^39^ In addition, the class I CPD protein CryA of *Aspergillus nidulans* was recently reported to fine-tune the oxidative stress response.^8^ However, whether Agaricomycetes class I CPD proteins are involved in the oxidative stress response remains to be clarified.

The losses of the class I CPD protein Phr1 in gasteroid species are in parallel with their loss in other eukaryotic lineages. Despite being one of the most ancient and most efficient UV-induced DNA damage repair pathways,^7^ photoreactivation has been lost in several lineages. A striking example is placental mammals, which no longer perform photoreactivation and instead rely on the less efficient and more energetically costly nucleotide excision repair pathway.^7^ The loss of photoreactivation in placental mammals was explained by the “nocturnal bottleneck hypothesis,”^40^ which posits that early placental mammals were in competition with dinosaurs and avoided daytime, which led to their adaptation to night life.^41^ Another notable parallel is the evolution of CPD photolyases in amphibians, where the “UV sensitivity of amphibians” hypothesis posits that there is a strong positive correlation between high photolyase levels and the hatching success of different species, suggesting that these enzymes contribute significantly to the UV-B resistance of the eggs.^42^ Indeed, it was recently verified that amphibians harboring CPD photolyases with functionally relevant, structurally disruptive amino acid variations have decreasing populations and that species without photolyases generally live in dark habitats.^43^ Our results provide a novel example of the loss of this remarkable protein family accompanying light-related lifestyle changes in distinct eukaryotic lineages. Overall, our analyses revealed that class I CPD photolyases are the most conserved CRY/PHR subfamily among mushroom-forming fungi. Within this clade, the repeated loss of class I CPDs is associated with the independent evolution of gasteroid fruiting body forms, providing an example of convergent genetic change accompanying fungal morphological evolution and of the genetic consequences of the secotioid syndrome. In addition, we demonstrated that Phr1 contributes to photoreactivation in the model pileate-stipitate mushroom, *C. cinerea*, providing clues on the putative function that became dispensable for gasteroid lineages. Together, these findings provide an example of how morphological and developmental innovations that may reduce exposure to DNA-damaging conditions could be associated with relaxed selective constraints on conserved molecular error-repair mechanisms, including class I CPD photolyases, leading to parallel gene losses in multiple related lineages.

### Limitations of the study

Although our genomic dataset tends to cover a broad fungal diversity, uneven taxonomic sampling and variable genome quality may have influenced the detection of CRY/PHR homologs, as well as ancestral copy number and gene loss inferences. In addition, our functional analyses were restricted to *C*. *cinerea*, limiting the extent to which the observed role of Phr1 in photoreactivation can be generalized across Agaricomycetes. While no obvious developmental phenotypes were detected under the tested conditions, subtle or context-dependent functions may have remained undetected. Also, we did not investigate the potential involvement of class I CPD proteins in oxidative stress-response-related processes, despite indications of such roles in other fungal groups.

## Resource availability

### Lead contact

Further information and requests for resources and reagents should be directed to and will be fulfilled by the lead contact, Zsolt Merényi (merenyi.zsolt@brc.hu).

### Materials availability

Plasmids (*Ccphr1*KO and *Ccphr1*OE) and mutant *Coprinopsis cinerea* strains generated in this study are available from the lead contact upon request.

### Data and code availability

• Genomic datasets analyzed during this study are publicly available at JGI (https://mycocosm.jgi.doe.gov/) and NCBI (www.ncbi.nlm.nih.gov).
• All other original data reported in this paper are available within the main text or the supplementary materials.
• The original code used for mapping results visualization is available on GitHub (https://github.com/zsmerenyi/compaRe) and ZENODO (https://doi.org/10.5281/zenodo.19691351).
• Any additional information required to reanalyze the data reported in this paper is available from the lead contact upon request.

## Acknowledgments

We thank Ildiko Unk for providing equipment for UV mutagenesis and Botond Hegedüs for providing data for analysis. This paper was supported by the János Bolyai Research Scholarship of the 10.13039/501100003825Hungarian Academy of Sciences (BO/00269/24/8 to Z.M.) and by the National Academy of Scientist Education Program of the National Biomedical Foundation under the sponsorship of the Hungarian Ministry of Culture and Innovation (to D.A.S.). We acknowledge the support by the 10.13039/501100011019National Research Development and Innovation Office (grant nos. 10.13039/501100003549OTKA 142188 and K-138022; Advanced 152158 and the Hungarian–Slovenian Research Cooperation Programme SNN25, grant no. 152010) and the 10.13039/501100000781European Research Council (grant no. 101086900 to L.G.N.).

## Author contributions

Conceptualization, M.V., Z.M., and L.G.N.; methodology, D.A.S., A.C., X.-B.L., D.N., A.V., M.V., and Z.M.; investigation, D.A.S., A.C., X.-B.L., D.N., A.V., M.V., and Z.M.; visualization, D.A.S., M.V., and Z.M.; funding acquisition, L.G.N.; project administration, M.V., Z.M., and L.G.N.; supervision, M.V., Z.M., and L.G.N.; writing – original draft, D.A.S., A.V., M.V., Z.M., and L.G.N; writing – review and editing, D.A.S., A.C., X.-B.L., A.V., M.V., Z.M., and L.G.N.

## Declaration of interests

The authors declare no competing interests.

## Declaration of generative AI and AI-assisted technologies in the writing process

During the preparation of this work, ChatGPT (OpenAI) was used to generate textual prompts for AI-based illustrations of fruiting bodies (*Guyanagaster*, *Cyathus*, *Rhizopogon*, *Melanogaster*, and *Pisolithus*) shown in Figure 2 and for grammatical corrections of the text. After using this tool, the authors have reviewed and take full responsibility for the content of the published article.

## STAR★Methods

### Key resources table

**Table:** 

REAGENT or RESOURCE	SOURCE	IDENTIFIER
**Bacterial and virus strains**		

*Escherichia coli* NEB5ɑ	New England Biolabs	Catalog #E5520S

**Chemicals, peptides, and recombinant proteins**		

Alt-R™ S.p. Cas9 Nuclease V3	Integrated DNA Technologies	Catalog # 1081058
Phusion Plus DNA Polymerase	Thermo Fisher Scientific	Catalog #: F630L
VinoTaste Pro	Novozymes	Catalog #: 23164
Phire Hot Start II PCR Master Mix	Thermo Scientific	Catalog #: F126L
PowerUp™ SYBR™ Green Master Mix for qPCR	Thermo Fisher Scientific	Catalog #: A25742

**Critical commercial assays**		

NucleoSpin Gel and PCR Cleanup Kit	Macherey Nagel	Catalog #:740609.50
NE Builder Hifi Assembly Cloning Kit	New England Biolabs	Catalog #: E5520S
Quick-RNA MiniPrep kit	Zymo Research	Catalog #: R1055
RevertAid First Strand cDNA Synthesis Kit	Thermo Fisher Scientific	Catalog #: K1622

**Deposited data**		

Protein files of 655 genomes	Mycocosm	https://mycocosm.jgi.doe.gov/
Reference CRY/PHR protein sequences	Mei and Dvornyk^4^	https://doi.org/10.1371/journal.pone.0135940
Illumina (QuantSeq) gene expression data	Hegedüs et al.^29^	GSE287886
RNA-Seq data of different developmental stages in *Schizophyllum commune* H4-8a and H4-8b	Krizsán et al.^44^	GSE125198
RNA-Seq data of different developmental stages in *Coprinopsis cinerea* #326	Krizsán et al.^44^	GSE125184
RNA-Seq data of different developmental stages in *Phanerochaete chrysosporium*	Krizsán et al.^44^	GSE125199
RNA-Seq data of different developmental stages in *Auriculariopsis ampla* NL-1724	Almasi et al.^45^	GSE132826
RNA-Seq data of different developmental stages in *Armillaria mellea* C18/9	Sipos et al.^46^	GSE100213
RNA-Seq data of different developmental stages in *Mycena kentingensis*	Ke et al.^47^	PRJNA623720
RNA-Seq data of different developmental stages in *Pterula gracilis*	Merenyi et al.^48^	GSE176181
RNA-Seq data of different developmental stages in *Pleurotus ostreatus* (N001)	Merenyi et al.^48^	GSE176181
RNA-Seq data of different developmental stages in *Lentinus tigrinus* RLP-9953-sp	Krizsán et al.^44^	GSE125190
RNA-Seq data of different developmental stages in *Lentinula edodes* Le (Bin) 0899 ss11 v1.0	Zhang et al.^49^	PRJNA649389
Fungal CRY/PHR protein sequences classified and analyzed in this study	This study	https://doi.org/10.5281/zenodo.19691351

**Experimental models: Organisms/strains**		

*Coprinopsis cinerea* Amut1Bmut1 #326 strain	N/A	FGSC#25122
*C. cinerea #326: A43mutB43mut pab1-1*	Swamy et al.^50^	N/A
*C. cinerea Δphr1: A43mutB43mut pab1-1 Δphr1::pab1-1*	this study	N/A
*C. cinerea Δdst1: A43mutB43mut pab1-1 Δdst1::pab1-1*	Provided by Xiao-Bin Liu	N/A
*C. cinerea phrOE: A43mutB43mut pab1-1 PgpdII::phr1::hph*	this study	N/A

**Oligonucleotides**		

Primers for plasmid constructions, see Table S4	Integrated DNA Technologies	N/A
Primers for checking	Integrated DNA Technologies	N/A
Primers for real-time qPCR	Integrated DNA Technologies	N/A
crRNA CGATTTCACACTTCGTAACC	Integrated DNA Technologies	N/A
tracrRNA	Integrated DNA Technologies	N/A

**Recombinant DNA**		

PMA412	Stanley et al.^51^	N/A
pUC19	New England Biolabs	Catalog #: E5520S
*Ccphr1*KO	this study	N/A
*Ccphr1*OE	this study	N/A

**Software and algorithms**		

R version (version 4.4.2)	R Development Core Team	https://www.r-project.org
IQ-TREE (version 2.3.2)	Minh et al.^52^	N/A
RAxML (version 8.2.12)	Stamatakis et al.^53^	https://github.com/stamatak/standard-RAxML
Mmseqs2 (version R15-6f462)	Steinegger and Söding^54^	https://github.com/soedinglab/MMseqs2
ContScout	Bálint et al.^55^	https://github.com/h836472/ContScout/
NCBI	NCBI	http://www.ncbi.nlm.nih.gov
ggplot2 (version 3.4.0)	Wickham et al.^56^	https://github.com/tidyverse/ggplot2
InterProScan (version 5.68–100)	Jones et al.^57^	https://github.com/ebi-pf-team/interproscan
MCL	Enright et al.^58^	https://micans.org/mcl/
MAFFT (version 7.525)	Katoh and Standley^58^	https://mafft.cbrc.jp/alignment/server/index.html
TrimAl (version 1.4)	Capella-Gutiérrez et al.^59^	https://vicfero.github.io/trimal/
NOTUNG (version 2.9)	Stolzer et al.^60^	https://www.cs.cmu.edu/∼durand/Notung/
COMPARE	Nagy et al.^61^	https://github.com/zsmerenyi/compaRe
BayesTraits (version 5.0.1)	Pagel^62^	https://www.evolution.reading.ac.uk/BayesTraitsV5.0.3/BayesTraitsV5.0.3.html
Script collection used for this work	This study	https://doi.org/10.5281/zenodo.19691351
SnapGene (version 4.3.11)	www.snapgene.com	RRID:SCR_015052

### Experimental model and study participant details

#### Strains and culture media

*C. cinerea* strains used in this study are listed in the key resources table. All strains were grown on YMG agar media (4 g/L yeast extract, 10 g/L malt extract, 4 g/L glucose, and 10 g/L agar) in the dark at 28°C and maintained at 4°C. Oidiation was carried out at 37°C under constant light on YMG agar medium, unless otherwise stated. Regeneration media was used for the regeneration of transformed protoplasts (2 g/L L-asparagine, 172 g/L sucrose, 5 g/L starch, 10 g/L agar, 4 g/L glucose, autoclaved separately, 25 mL stock A, 1 mL stock B, 10 mL stock C added after autoclaving). The transformant cultures were maintained on minimal media (2 g/L L-asparagine, 10 g/L agar, 10 g/L glucose, autoclaved separately, 25 mL stock A, 1 mL stock B, 10 mL stock C added after autoclaving, containing 100 μg/mL hygromycin and 100 μg/mL *para*-aminobenzoic acid in case of overexpressing strains). Stock solutions: A (40 g/L KH_2_PO_4_, 90 g/L Na_2_HPO_4_, 11.6 g/L Na_2_SO_4_, 20 g/L di-ammonium tartrate, distilled water), B (40 mg/L thiamine, distilled water), C (25 g/L MgSO_4_ x 7 H_2_O distilled water). *Escherichia coli* NEB5ɑ was used for plasmid amplification and maintenance. *E. coli* strains were grown in liquid LB medium (10 g/L tryptone, 2 g/L yeast extract, 10 g/L NaCl) at 37°C with agitation (220 rpm).

### Method details

#### Dataset and identification of cryptochromes

A total of 655 genomes (referred to as the G655 dataset) were downloaded from JGI (https://mycocosm.jgi.doe.gov/) and NCBI (www.ncbi.nlm.nih.gov) before October 22, 2024 (Table S1). This dataset includes 12 non-fungal Holozoa and Holomycota species used as outgroups, 62 non-Dikarya fungi, 235 Ascomycota, and 346 Basidiomycota.

Cryptochromes were identified in these genomes, using a reference dataset consisting of 499 cryptochromes representing all major divisions of life, as compiled by Mei and Dvornyk.^4^ Sequence similarity searches between reference cryptochromes and the G655 dataset were performed using MMseqs2^54^ in ultrasensitive search mode (-s 7.5) to detect distant homologs. Three iterative search cycles (--num-iterations 3) were conducted to enable profile-based searching. Significant hits were defined using an E-value threshold of 0.0001 (-e 0.0001) and a minimum of 10% query coverage (-c 0.1; --cov-mode 0). A maximum of 5,000 target sequences per query (--max-seqs 5000) were retained. This initial MMSeqs search yielded 5,020 protein sequences without any filtering.

These 5,020 proteins were further analyzed using InterProScan (version 5.68–100)^57^ and subjected to all-vs-all similarity searches. The results of this second MMseqs2 analysis were filtered using bidirectional coverage thresholds of 0%, 25%, and 50%, followed by MCL^58^ clustering to generate protein clusters. Manual inspection, incorporating the top hits from the initial search, the clustering results, and domain content analysis, revealed that many false-positive hits among the initial 5020 proteins were due to improper queries (e.g., fused proteins). After removing these obvious false positive hits, 1,128 cryptochromes were confidently identified.

A gene tree was constructed from these 1,128 sequences. Sequences were aligned using the MAFFT v7.525^63^ auto algorithm, trimmed using TrimAl v1.4^59^ with the strict method, and a phylogenetic tree was generated using a maximum likelihood approach in IQ-TREE v2.3.2^52^ with automatic model selection and 1,000 ultrafast bootstrap replicates. The CRY/PHR gene tree was also generated using RAxML v8.2.13^53^ with the bootstrap and best-scoring ML tree search option, applying the PROTGAMMALG substitution model and 100 bootstrap replicates. Based on homology search results and the reconstructed gene tree, all identified cryptochromes were classified into five well-known subfamilies: class I CPD, class II CPD, CRY-DASH, (6-4)-PHR, and Plant-CRY, following the classification of Mei and Dvornyk.^4^ The (6-4)-PHR and animal CRY groups were merged into a single (6-4)-PHR clade, as they formed one cluster in Figure 2 of Mei and Dvornyk.^4^ Plant PHR2 was not detected in fungi. Classified protein IDs are provided in Table S2.

To identify the origin of fungal plant CRYs, we performed NCBI BLAST searches using CRY/PHR sequences from *Malassezia* as queries. The best BLAST hits were retrieved and used to reconstruct gene trees. Two independent multiple sequence alignments were generated: one using PRANK^64^ and the other using MAFFT with the L-INS-i algorithm. Both alignments were trimmed with TrimAl using the gappyout option. Maximum-likelihood phylogenetic trees were inferred from each trimmed alignment using RaxML v8.2.13 under the PROTGAMMALG model with 100 bootstrap replicates. To exclude the possibility of potential genomic contamination, we screened the Malassezia genomes using ContScout,^55^ which did not flag these sequences as contaminants.

#### Species tree reconstruction

For species tree inference of the G655 dataset, as a starting point we identified the 277 marker genes used in Szánthó et al.,^65^ and we reconstructed the phylogenetic tree as described in Wu et al.^66^ Briefly, multiple sequence alignments were performed using the MAFFT L-INS-i algorithm and trimmed using TrimAl v1.4 with the strict option. Alignments shorter than 150 amino acids or containing fewer than 500 species were discarded, resulting in 221 single-copy clusters comprising 74,376 aligned sites used for tree reconstruction. Phylogenetic inference was performed using IQ-TREE v2.3.2 with the LG + G substitution model and 1,000 ultrafast bootstrap replicates.

#### Gene tree reconstruction and gene family evolution

As the divergence of cryptochrome subfamilies predates the emergence of Holomycota (Figure S1), gene family evolution was evaluated separately for each subfamily. To this end, sequences from each subfamily were aligned using MAFFT L-INS-i and trimmed with TrimAl v1.4 (-gt 0.2). Phylogenetic trees were reconstructed using IQ-TREE with automatic model selection and 1,000 ultrafast bootstrap replicates. Rooting and reconciliation of gene trees with the species tree were conducted using NOTUNG v2.9,^60^ with an edge-weight threshold of 90. Gene duplication and loss histories were inferred using Dollo parsimony, implemented in a modified version of COMPARE.^61^ Mapping results were visualized using a custom R script (https://github.com/zsmerenyi/compaRe), following the method described by Merényi et al.^67^

To assess the relationship between recent copy numbers of class I CPD cryptochromes and morphological traits, all Basidiomycota species in the G655 dataset were manually classified and coded binary according to their most complex morphological structures (e.g., fruiting body types following the categories used by Varga et al.^68^ and Virágh et al.^18^; see Table S1). We examined the potential correlated evolution between the presence/absence of class I CPD genes and the above-mentioned morphological trait using a continuous-time Markov model implemented in BayesTraits.^62^^,^^69^

For this analysis, a species tree was constructed using the same genetic markers as in the full G655 dataset, but limited to the Basidiomycota subset (G347 dataset). Phylogenetic inference was performed using IQ-TREE with an ultrafast bootstrap analysis comprising 1000 replicates. From these, 100 trees were randomly selected for use in the DISCRETE module of BayesTraits. Model fit was evaluated using a likelihood ratio (LR) test, calculated as LR = −2 × (log likelihood of the independent model − log likelihood of the dependent model). The LR statistic was interpreted following the methodology described by Barker and Pagel.^70^

#### Design and *in vitro* assembly of the ribonucleoprotein (RNP) complex

crRNA was designed with the software sgRNAcas9^71^ using the genomic regions of the *phr1* gene of *C. cinerea* #326 *A43mutB43mut pab1-1* (protein ID: CopciAB_492962; protospacer: CGATTTCACACTTCGTAACC). For *in vitro* assembly of the ribonucleoprotein complex, 1.2 μL of synthetic crRNA (100 μM), 1.2 μL of synthetic tracrRNA (100 μM) (Integrated DNA Technologies), and 9.6 μL of duplex buffer (Integrated DNA Technologies) were mixed and incubated for 5 min at 95°C and cooled to room temperature. Then 0.5 μL purified Alt-R S.p. Cas9 Nuclease V3 (Integrated DNA Technologies), 1 μL duplex buffer, and 1.5 μL Cas9 reaction buffer,^72^ were added to the mixture, followed by an incubation for 15 min at 37°C. The assembled RNP-complex was stored on ice until use.

#### Plasmid construction

Oligonucleotides used for plasmid construction are listed in Table S4. Construction of *Ccphr1*KO was carried out using the NE Builder HiFi Assembly Kit (New England Biolabs). To this end, three fragments with overlapping overhangs were amplified: (1) a 1000 bp long upstream homology arm with its 3′ end located 10 bp away upstream from the cut site of the Cas9 protein on the *phr1* protospacer region from the genome of *C. cinerea* #326, (2) the wild-type *para*-aminobenzoic acid synthase (*paba*) gene (used as selection marker) that was amplified with its native promoter and terminator region from the plasmid PMA412^51^ and (3) a 1000 bp long downstream homology arm with its 5′ end located 10 bp away downstream of the cut site of the Cas9 cut site on the *phr1* protospacer region from the genome of *C. cinerea* #326. Overlapping primers were designed using Snapgene’s Gibson Assembly function (version 4.3.11). All fragments used in the vector construction were amplified using Phusion Plus DNA Polymerase (Thermo Fisher Scientific), following the manufacturer’s instructions. Amplicons were purified using the NucleoSpin Gel and PCR cleanup Kit (Macherey-Nagel). Purified fragments were then assembled with the PCR-linearized pUC19 backbone according to the instructions of the NE Builder Hifi Assembly Cloning Kit (New England Biolabs). To construct the plasmid *Ccphr1*OE, three fragments were amplified: (1) the *E. coli* hygromycin B phosphotransferase gene (used as a selection marker) with the *C. cinerea* β-tubulin promoter and terminator from the plasmid that was assembled previously in our lab,^73^ (2) the *Agaricus bisporus gpd*II promoter from the plasmid PMA412, and (3) the *phr1* ORF along with its native 1000 bp terminator region. These fragments were also assembled using the NEBuilder Hifi Assembly Cloning Kit along with the PCR-linearized pUC19 backbone.

#### Transformation of *C. cinerea*

For the transformation of *C. cinerea*, protoplasts made out of germinating oidia were used. To germinate oidia, an oidia suspension of a concentration of 10^8^ oidia/ml was inoculated on YMG plates covered with cellophane using a multichannel pipette by touching the cellophane with the tip of the pipette across the plate. Next, colonies were grown for 18 h at 37°C in the dark. The next day, a 2× PP buffer (for 25 mL: 13.7 mL 2M KCL; 6.4 mL 1.1M KOH; 480 mg citric acid; 3.2g VinoTaste Pro (Novozymes); 3.0 mL ddH_2_0) was prepared, filter-sterilized, and diluted to 1× with sterile liquid YMG. Using sterile forceps, the germinating oidia were harvested into the 1× PP buffer and incubated at 30°C for 3 h with slight agitation at 70 rpm. The protoplasts were then centrifuged at 1000 g for 10 min at 4°C and resuspended in 5 mL of ice-cold STC buffer (1.2M sorbitol, 10 mM Tris pH7.5, 10 mM CaCl_2_), centrifuged again, and then resuspended in ice-cold STC buffer (100 μL/number of transformation reactions). 25 μL of ice-cold PEG/CaCl_2_, 10 μg of pab1+ DNA, and the 15 μL of the RNP complex were mixed with 100 μL of protoplast suspension and incubated on ice for 30 min. Then, 500 μL of PEG/CaCl_2_ was added to the reaction and incubated at room temperature for 5 min 1 mL of STC and 20 mL of top agar (regeneration media supplemented with 0.6% agar) were added, and the mixture was divided into 4 and poured on the surface of regeneration media agar plates without PABA. The plates were incubated at 37°C in the dark, and the colonies appeared after 3–4 days.

#### Transformant screening

The appearing colonies after transformation were collected on minimal plates. To screen the transformants, a fast, microwave-based DNA extraction method^74^ was used to prepare template DNA for PCR. Briefly, a pinhead sample of mycelium (ca. 4 mm^2^) was scraped from the surface of plates and transferred into a 1.5 mL sterile microcentrifuge tube containing 100 μL of sterile distilled water. The closed tubes were microwaved for 1 min at 600 W in a microwave oven, shortly vortexed, stored for 30 s at room temperature, and microwaved again for 1 min at 600 W. Afterward, tubes were transferred for about 10 min to −20°C and then centrifuged at 9300 g for 5 min 0.5 μL of resulting supernatant was directly used as a template in a 20 μL PCR reaction. For the screening PCR, the primer pair Ccphr_check.F and Ccphr_check.R (sequences are listed in Table S4) and Phire Hot Start II PCR Master Mix (Thermo Scientific) were used according to the manufacturer’s instructions. Isolation of pure strains was conducted by obtaining single spore colonies by first oidiating the selected strains, then diluting the harvested oidia suspension and inoculating ∼100 oidia onto minimal media (supplemented with 100 μg/mL hygromycin and 100 μg/mL of PABA if needed), then separating the appearing colonies on minimal media.

#### Examination of vegetative growth

To examine the vegetative growth speed, mycelial plugs (d = 5 mm) from each strain were inoculated on YMG medium and grown for 96 h in the dark, under white, monochromatic red (λ_max_ = 667 nm, 40 μmol m^−2^ s^−1^) and blue (λ_max_ = 470 nm, 40 μmol m^−2^ s^−1^) light at 28°C. The colony diameters were measured every 24 h.

#### Assessing oidia production

To quantify oidia production across wild type and mutant strains under various light conditions, strains were inoculated (d = 5 mm mycelial plugs) to YMG (0.4% glucose) plates, and cultured at 28°C for 3 days in the dark, then placed to constant white (Panasonic FL4055 ENW/37, 40 W, 37 mm tubes, 25 μmol m^−2^ s^−1^), monochromatic blue (λmax = 470 nm, 40 μmol m^−2^ s^−1^) and red (λmax = 667 nm, 40 μmol m^−2^ s^−1^) light, or left in the dark for 48 h at 28°C. The colony diameters were measured, and the oidia were harvested into distilled water (10–15 mL/plate) by scraping the surface of the colony with a blunt spatula and then filtered through a cell strainer (40 μm pore diameter) to get rid of hyphal remnants. The oidia suspension was centrifuged at 2400 g for 5 min, and the oidia were resuspended in distilled water, then counted under a Bürker chamber.

#### RNA purification and real-time qPCR

To test the effect of different wavelengths of light on the expression of *phr1* and *dst1*, in the wild type and the mutants, strains were cultured on YMG + cellophane in the dark at 28°C for 72 h, and then placed under constant white (Panasonic FL4055 ENW/37, 40 W, 37 mm tubes, 25 μmol m^−2^ s^−1^), monochromatic blue (λmax = 470 nm, 40 μmol m^−2^ s^−1^) or red (λmax = 667 nm, 40 μmol m^−2^ s^−1^) light for 2 h, or remained in the dark at 28°C. After 2 h of light exposure, equal amounts of mycelia (half of the colonies, ∼170 mg) were harvested from the plates and placed into liquid nitrogen. RNA was extracted using the Quick-RNA MiniPrep kit (Zymo Research), following the manufacturer’s instructions. Reverse transcription was carried out using the RevertAid First Strand cDNA Synthesis Kit (Thermo Fisher Scientific) according to the manufacturer’s instructions. real-time qPCR was performed using a Bio-Rad Opus 96X machine (Bio-Rad) and the PowerUp SYBR Green Master Mix for qPCR (Thermo Fisher Scientific). Cycling conditions: 95°C 2 min, followed by 40 cycles of 95°C 15 s, 60°C 1 min, and melt curve analysis. The *C. cinerea* β-tubulin gene (JGI Mycocosm protein ID: 393528) was used as a reference gene. For the determination of the relative expression of the *phr1* gene, the dark-treated WT strain was used as a standard. To test the effect of the deletions/overexpression of *phr1* and *dst1* genes under different wavelengths of light, the WT strain under the respective wavelength was used as a control. Expression changes were calculated using the formula FC = 2−ΔΔCt.^75^ Primers used and product lengths are listed in Table S4.

#### Photoreactivation assessment

To assess photoreactivation, we observed the effect of UV on the survival rate of the oidia of the wild type and the Δ*phr1* strain. To determine the UV susceptibility of *C. cinerea,* 500 oidia/plate were spread on YMG agar plates and incubated in the dark for 3 h at 28°C. Next, plates were exposed to different UV-doses (0–100 J/m^2^) and immediately moved to the dark at 28°C for two days. Then, colony-forming units (CFUs) were counted, and the obtained values after different UV-doses were compared to the untreated control. Next, we observed the effect of light treatment on the survival rate of the oidia of the WT and the Δ*phr1* strain after a UV dose of 60 J/m^2^. To this end, oidia were handled similarly to the susceptibility test, except that after UV exposure, half the plates were moved to the dark immediately, the other half were incubated under white light (INESA LED Tri-proof light MSF426LED18W, 18 W) for 1 h, and then moved to the dark at 28°C. CFUs were counted after 2 days.

#### Fruiting body production

To obtain fruiting bodies, 500 mL beakers were filled with 100 mL YMG (0.6% agar, 0.2% glucose). A cube (approx. 1 × 1 × 1 cm) of water agar complemented with sulfates (1% agar, 0.2 mM CuSO_4_, 0.2 mM MgSO_4_) was placed on one side of the beaker. *C. cinerea* strains (d = 5 mm mycelial plugs) were inoculated, and incubated for 2 days at 37°C in the dark, then transferred to 28°C to 12-h light/12-h dark periods with ∼80% relative humidity until maturation.

### Quantification and statistical analysis

All statistical analyses were conducted in the R environment. Data are typically presented as mean ± standard deviation (SD) or as median with interquartile range (IQR). Differences between groups were determined using the Wilcoxon signed-rank test for photoreactivation assays, and one-sample *t* test for oidia production comparisons. Statistical significance was defined as *p* < 0.05. The exact value of n, representing the number of independent biological replicates, the number of technical replicates (k), and the specific *p*-values are detailed in the corresponding figure legends. For RT-qPCR, expression changes were calculated using the FC = 2^−ΔΔCt^ method,^75^ with Ct values filtered for SDCt <0.5. Survival rates (SR) after UV-treatment were calculated by dividing the CFU count of the UV-treated samples (incubated either in the dark or under light) by the CFU count of the untreated control: $SR_{dark}=\frac{CFU_{UV60,dark}}{CFU_{untreated,dark}}$, $SR_{light}=\frac{CFU_{UV60},light}{CFU_{untreated,dark}}$ Then, light/dark survival ratio was obtained by dividing SR_light_ by SR_dark_.

## Supplemental information

Document S1. Figures S1–S13

Table S1. Metadata of the genomes used in the analysis

Table S2. List of the identified CRY/PHR genes

Table S3. List of *C. cinerea* homologs that were parallely lost in gasteroid lineages

Table S4. List of primers used in this study
