# Supplementary material for: Basidiomycete fruiting body morphology correlates with the convergent loss of photolyase-related proteins
Source: iScience. 2026 Aug 19;29(9):117265. doi: 10.1016/j.isci.2026.117265 (PMC13521097; doi:10.1016/j.isci.2026.117265)

**iScience, Volume *29***

## Supplemental information

# Basidiomycete fruiting body morphology correlates with the convergent loss of photolyase-related proteins

**Máté Virágh, Dorottya Anna Szafián, Árpád Csernetics, Xiao-Bin Liu, Dóra Nyári, András Viczián, László G. Nagy, and Zsolt Merényi**

**Figure S1: Gene Tree of CRY/PHR family.** Colored reference cryptochromes and photolyase-related proteins (CRY/PHR) originate from the work of Mei and Dvornyk[3], which we used as a basis for classifying fungal CRY/PHRs (black tip labels). For better visualization, we selected and displayed only 55 Holomycota lineages, which together cover all types of fungal CRY/PHR family members. The gene tree constructed from all fungal CRY/PHR family sequences analyzed in this study is shown in Figure S2. The maximum-likelihood phylogenetic tree was inferred using RAxML under the LG + Γ (PROTGAMMALG) model, with node support values in the nodes representing bootstrap percentages from 100 replicates, and the scale bar indicates the number of amino acid substitutions per site.

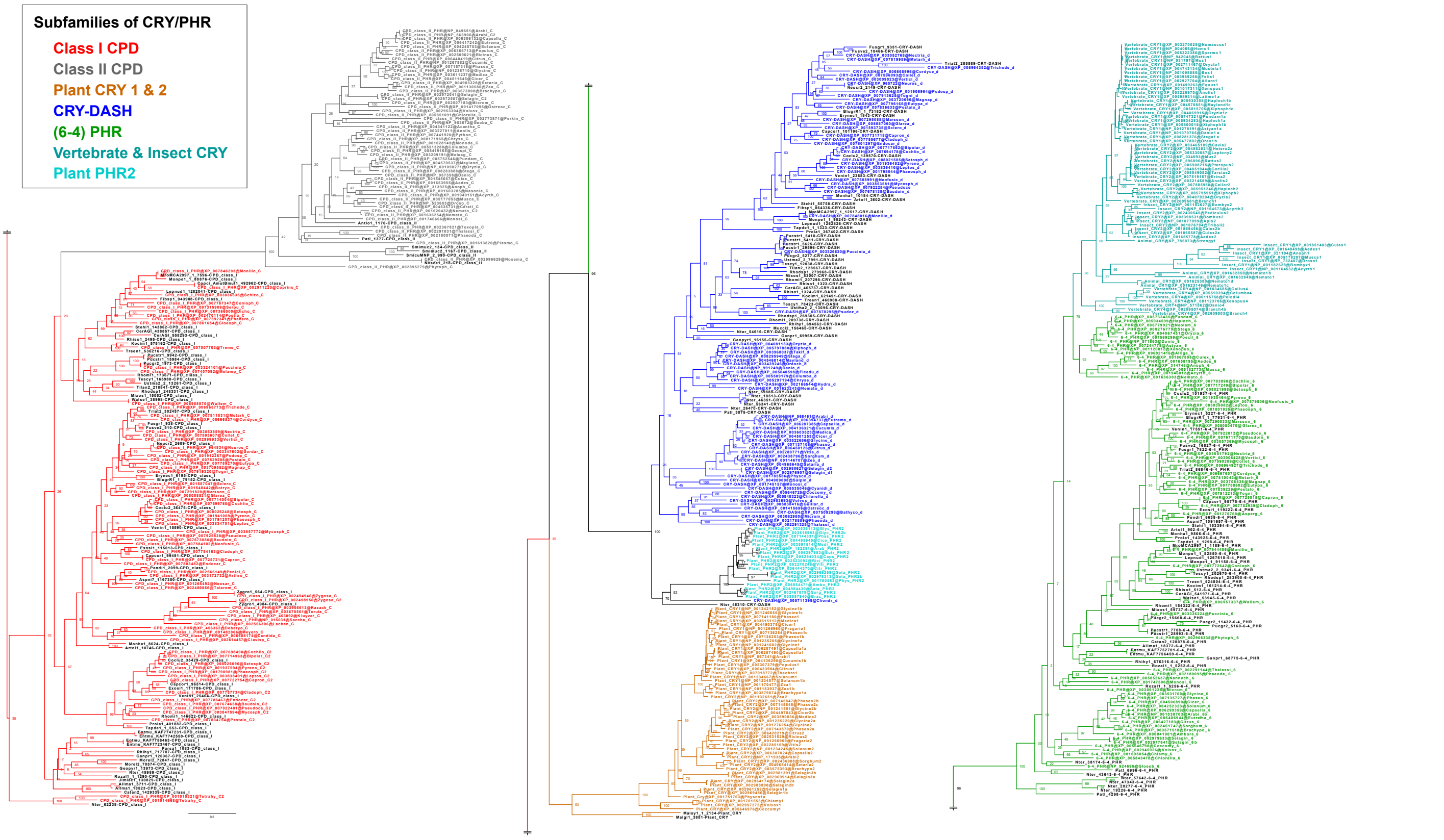

**Figure S2: Gene Tree containing the fungal members of the CRY/PHR family.** The gene tree shows 1128 members of the Fungal CRY/PHR gene family identified and classified among 655 genomes. The maximum-likelihood phylogenetic tree was inferred using RAxML under the LG + Γ (PROTGAMMALG) model, with node support values in the nodes representing bootstrap percentages from 100 replicates, and the scale bar indicating the number of amino acid substitutions per site.

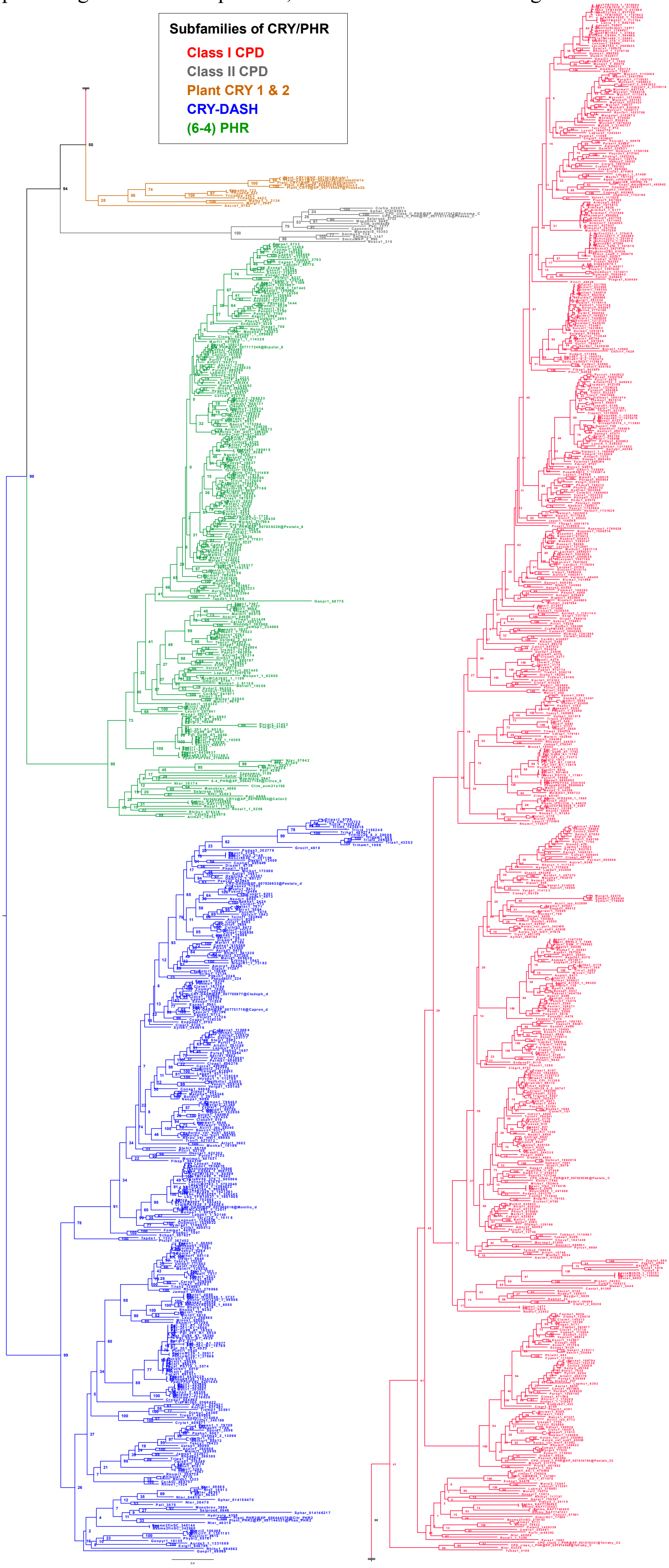

**Figure S3: Net gain reconstruction for CRY/PHR subfamilies.** The reconstruction implies that even the most abundant subfamilies of the CRY/PHR family tend to show frequent losses (red circles) across the fungal tree of life and only a few, sometimes species-specific, duplications (gain, with blue circles) without considerable expansion. The size of circles represents the inferred number of events.

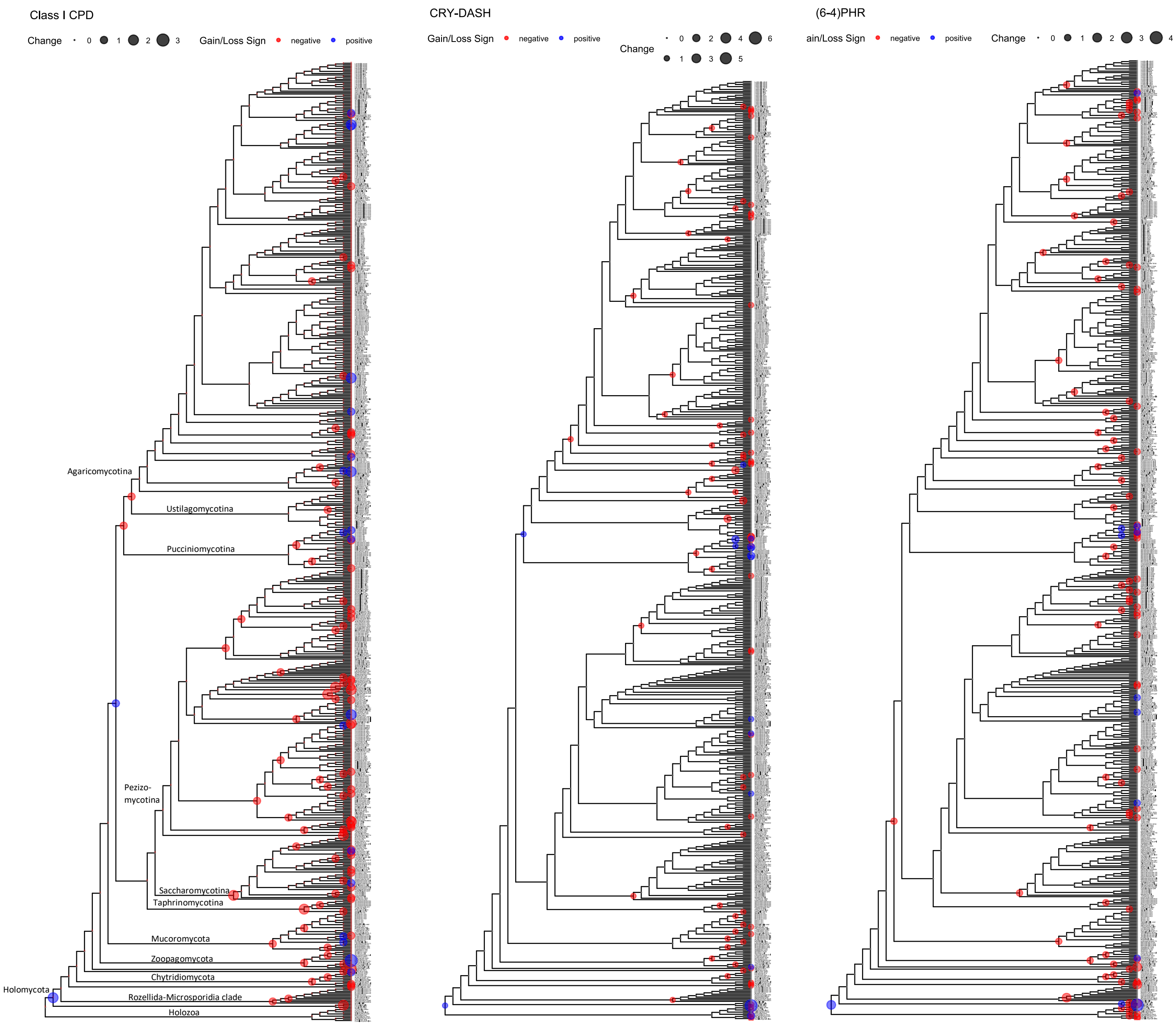

**Figure S4: Copy number reconstruction for CRY/PHR subfamilies.** While Class I CPD shows a conserved occurrence along the fungal tree of life, CRY-DASH and (6-4) PHR subfamilies are generally lost from e.g. Agaricomycotina. Separate phylogenetic trees are shown for each subfamily, and blue circle size represents the inferred number of gene copies in each subfamily.

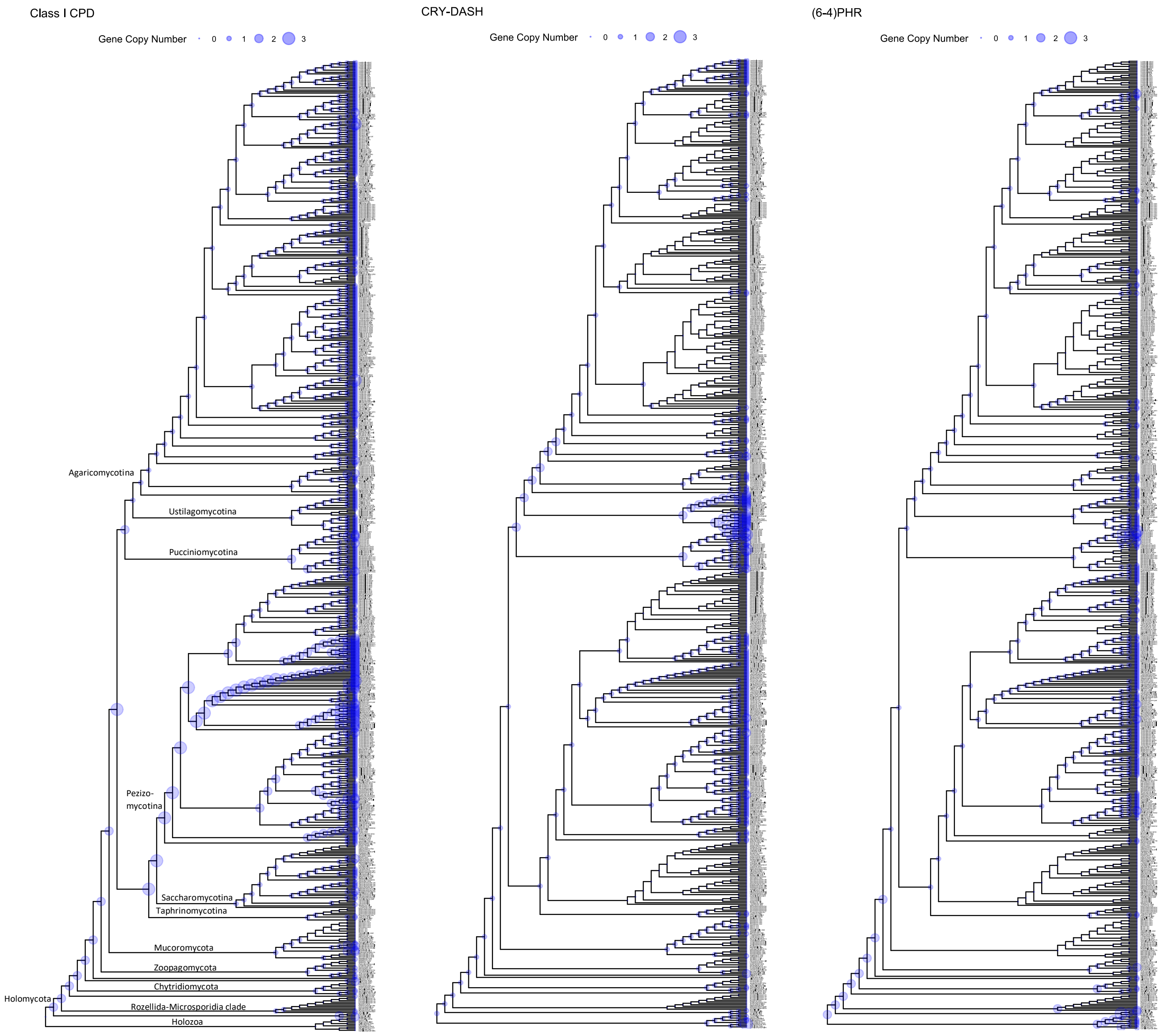

**Figure S5: Gene tree of the Plant CRY subfamily.**

Phylogenetic reconstruction of the Plant CRY subfamily (CRY/PHR gene family) suggests horizontal gene transfer from prokaryotes to the *Malassezia* clade (Phylum Basidiomycota) and the *Trichoplax* clade (Phylum Placozoa). Both maximum-likelihood phylogenetic trees were inferred using RAxML under the LG + Γ (PROTGAMMALG) model, with node support values in the nodes representing bootstrap percentages from 100 replicates. We applied different alignments for the two reconstructions A) PRANK alignment B) MAFFT L-INS-I alignment, and each alignment was trimmed with TrimAL using the gappyout algorithm. Some representatives of class I CPD genes were selected as outgroup. The *Trichoplax* and *Malassezia* clades are highlighted in blue and magenta, respectively. Taxon names and accession numbers were obtained from NCBI. The scale bar indicates the number of amino acid substitutions per site.

A

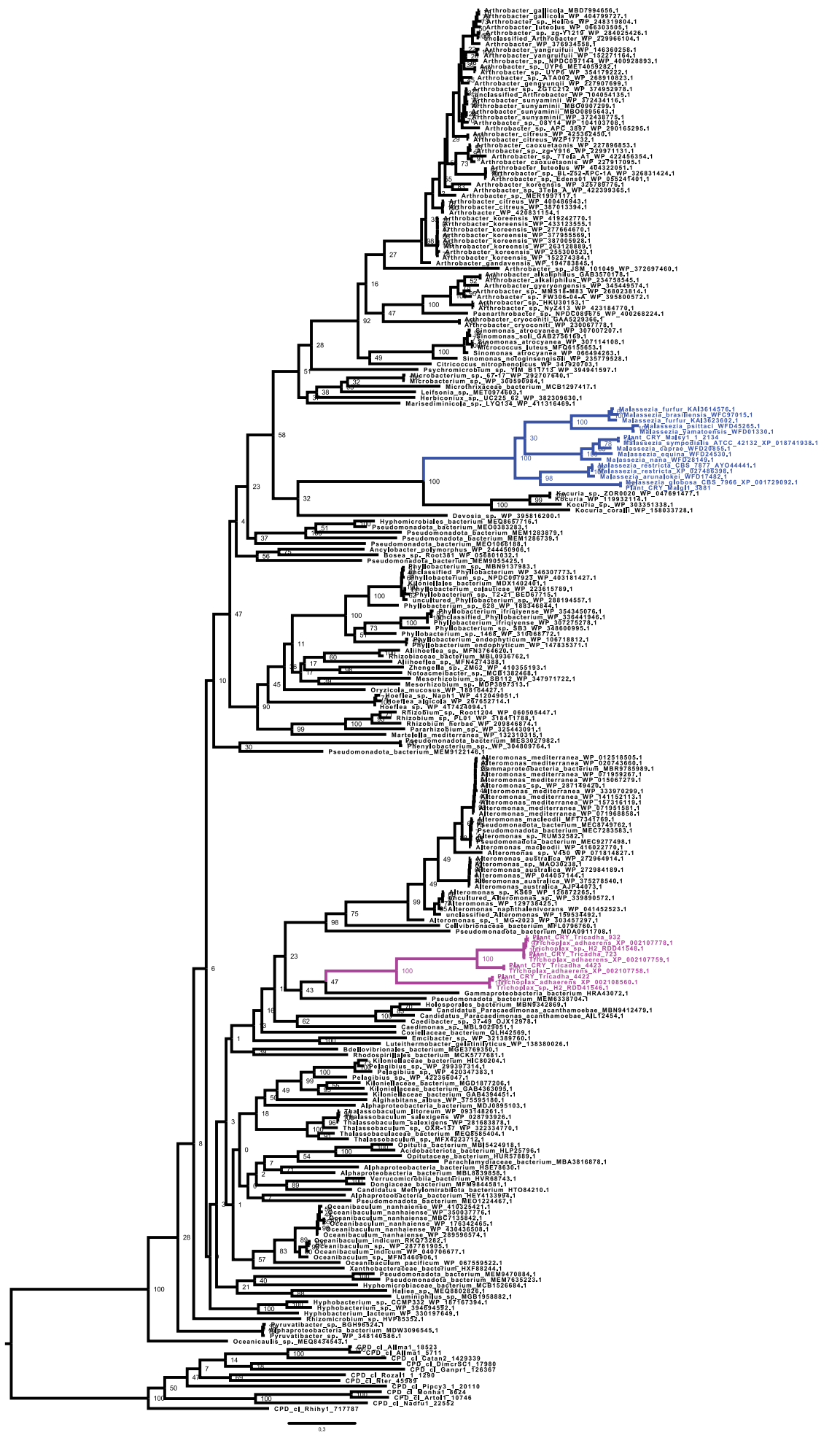

B

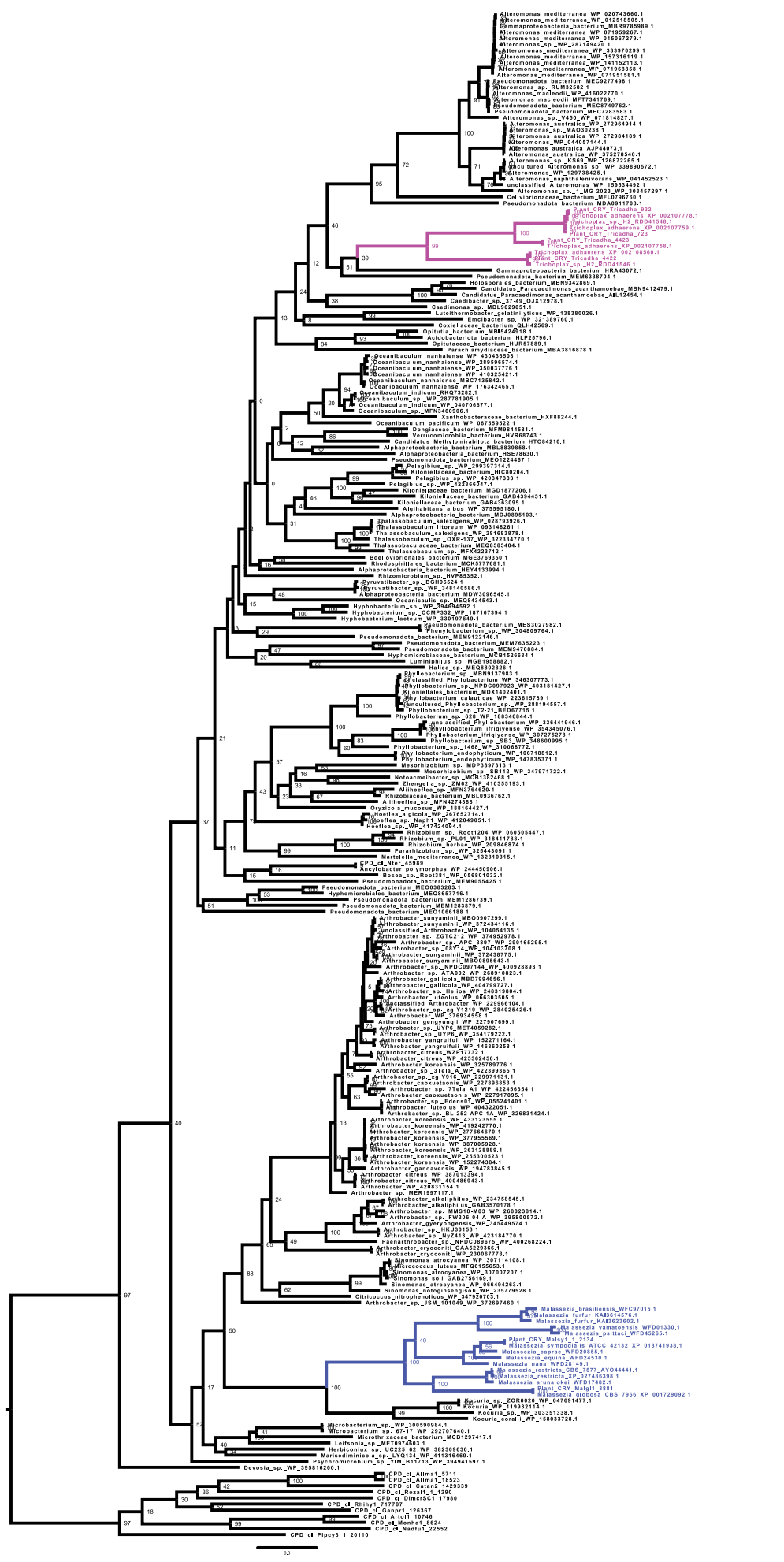

**Figure S6: Diversity loss of CRY/PHR family in Basidiomycota**

Species phylogeny of Basidiomycota showing the inferred evolution of class I CPD, CRY-DASH, and (6-4)-PHR subfamilies. The most recent common ancestor of Basidiomycota possessed members of all three subfamilies (darkest grey branches). Subsequent lineage-specific losses, particularly of CRY-DASH (red square) and (6-4)-PHR (green triangle), contributed to the reduced diversity of the CRY/PHR family, especially within Agaricomycetes. Although class I CPDs are the most prevalent (blue branches), they have also undergone losses (blue circle), most notably in lineages with gasteroid fruiting body morphologies (red rectangles in column morphology), where spores develop inside enclosed fruiting structures. Branch thickness indicates the number of subfamilies present, while branch color represents subfamily composition. For better visualization, branches containing only class I CPD are shown in blue, and branches lacking all CRY/PHR are shown in red. Symbols indicate complete loss of individual subfamilies: blue circles for class I CPD, red squares for CRY-DASH, and green triangles for (6-4)-PHR. Circles in tip labels represent the presence (filled) and absence (empty) of each CRY/PHR subfamily in recent genomes.

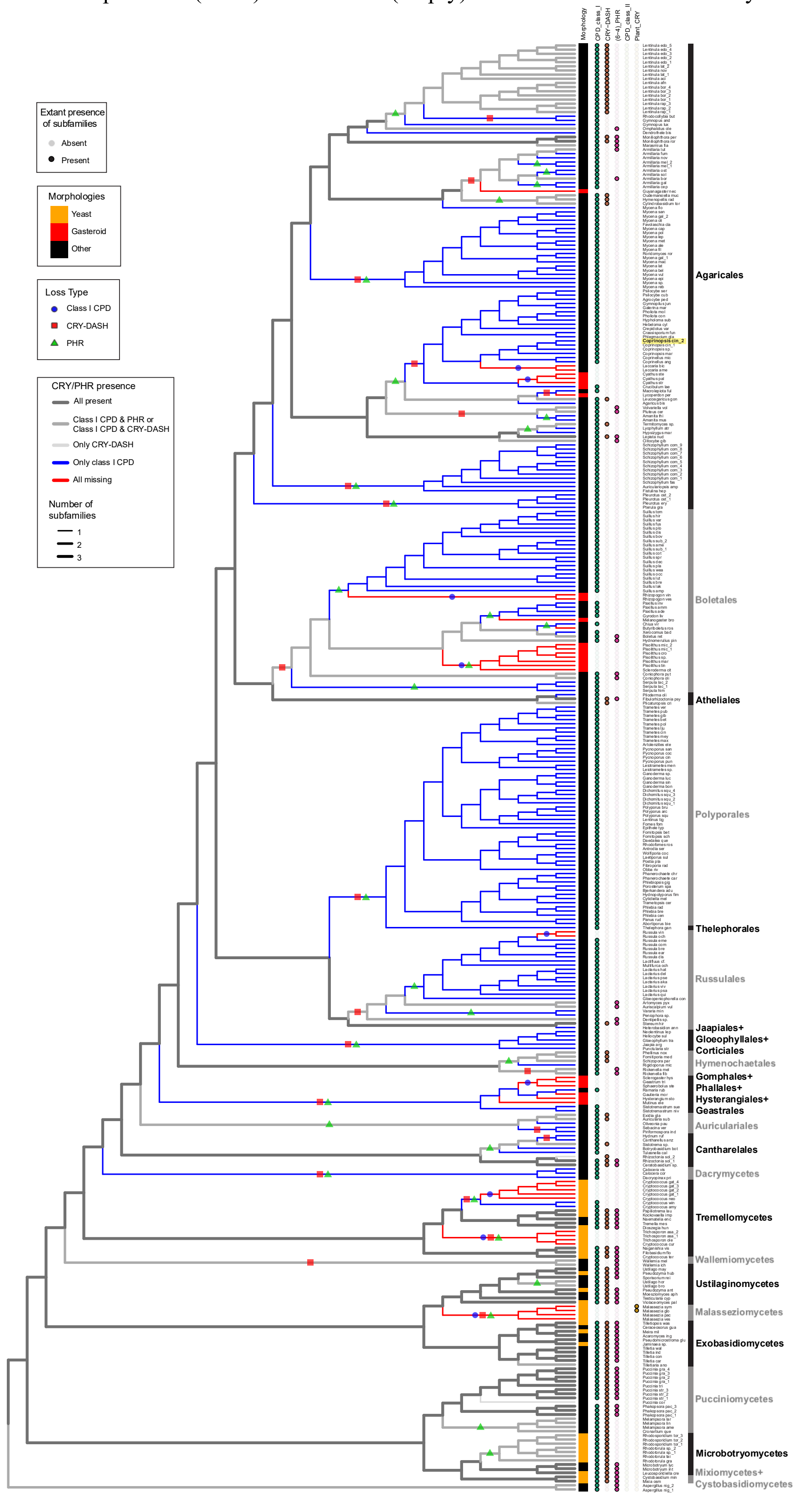

**Figure S7: Supplementary Figure 7. Validation of the disruption and overexpression of *phr1* in *C. cinerea.* A:** Schematic figure of the insertion of the para-aminobenzoic acid synthase gene in the first exon of the Phr1-encoding gene and its validation by Sanger sequencing in two independent mutants (Δ*phr1.1*, Δ*phr1.2*). **B:** Validation of the lack of expression of *phr1* in the Δ*phr1.1*, selected for downstream analysis, by RT-qPCR (n=3, k=2), **: p<0.005, One-sample t-test, dashed red line represents baseline calculated from the expression of *phr1* in the WT strain in the dark. **C:** Validation of the overexpression of *phr1* in the dark (n=3, k=2) and under monochromatic blue light (n=4, k=2) in the phr1 overexpression background compared to the expression of phr1 in the WT under the respective conditions. *: $p < 0.05$; **: $p < 0.005$; ***: $p < 0.0005$, one-sample t-test.

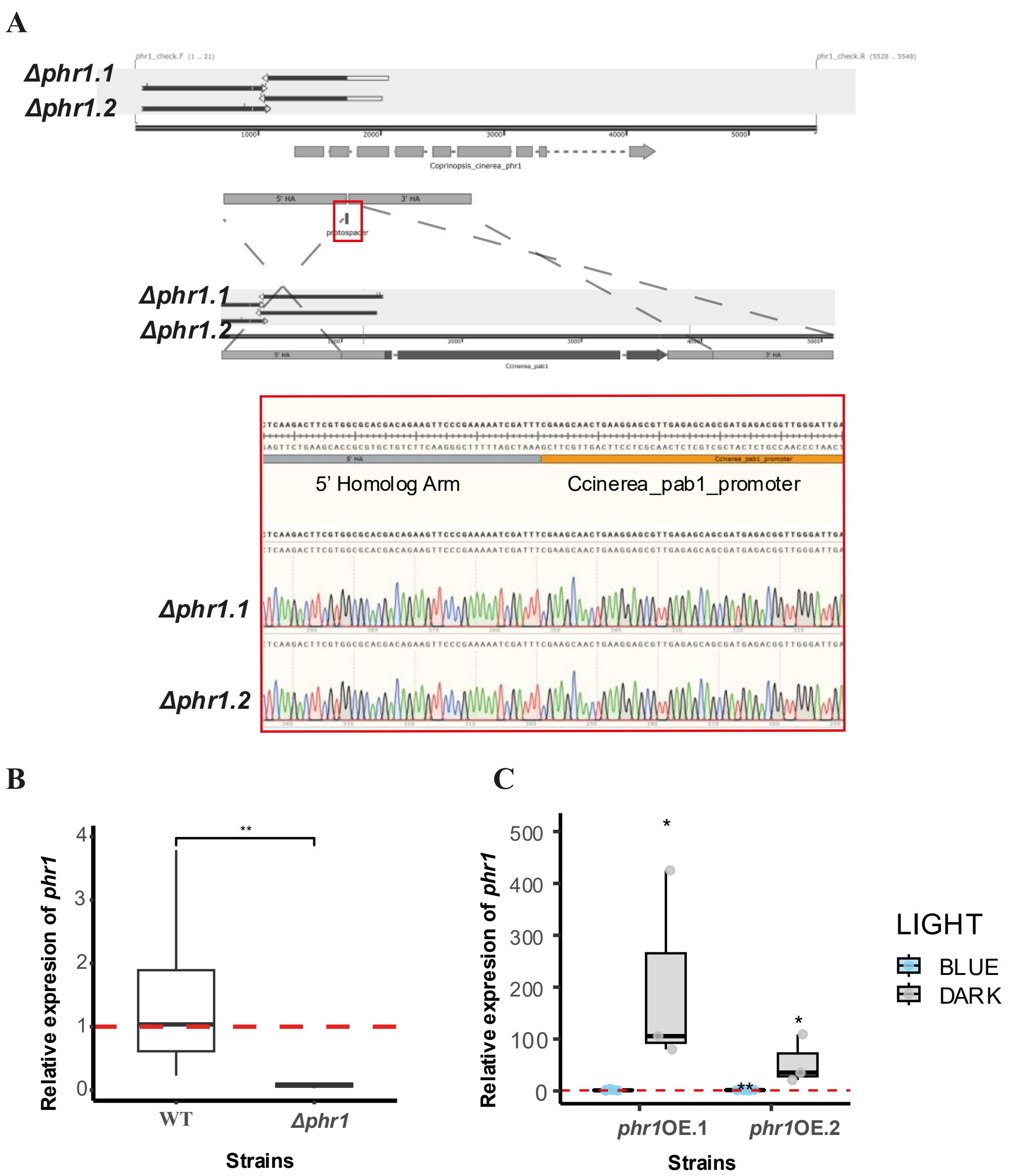

**Figure S8: Phr1 contributes to photoreactivation in *C. cinerea in vivo*.** To assess the role of *C. cinerea* Phr1 in photoreactivation, we determined the light/dark survival ratio of both the WT and the two phr1 disruptant strains (Δ*phr1.1 and* Δ*phr1.2*) after UV exposure. Approximately 500 oidia were plated onto YMG agar medium and subjected to a UV dose of 60 J/$m^2$. After irradiation, plates were either subjected to one hour of light treatment before incubation in the dark or moved immediately to the dark at 28°C for 48 hours. The light/dark survival ratio was determined for the wild type and Δ*phr1* strains by dividing the survival rate of UV-exposed spores after light treatment by the survival rate without light treatment. Each point on the IQR box plots represents the light/dark survival ratio from one of six biological replicates (n = 6), with each biological replicate calculated as the mean of five technical replicates (k=5). Statistical significance was assessed using a Wilcoxon signed-rank test; *: $p < 0.05$; **: $p < 0.005$; ***: $p < 0.0005$.

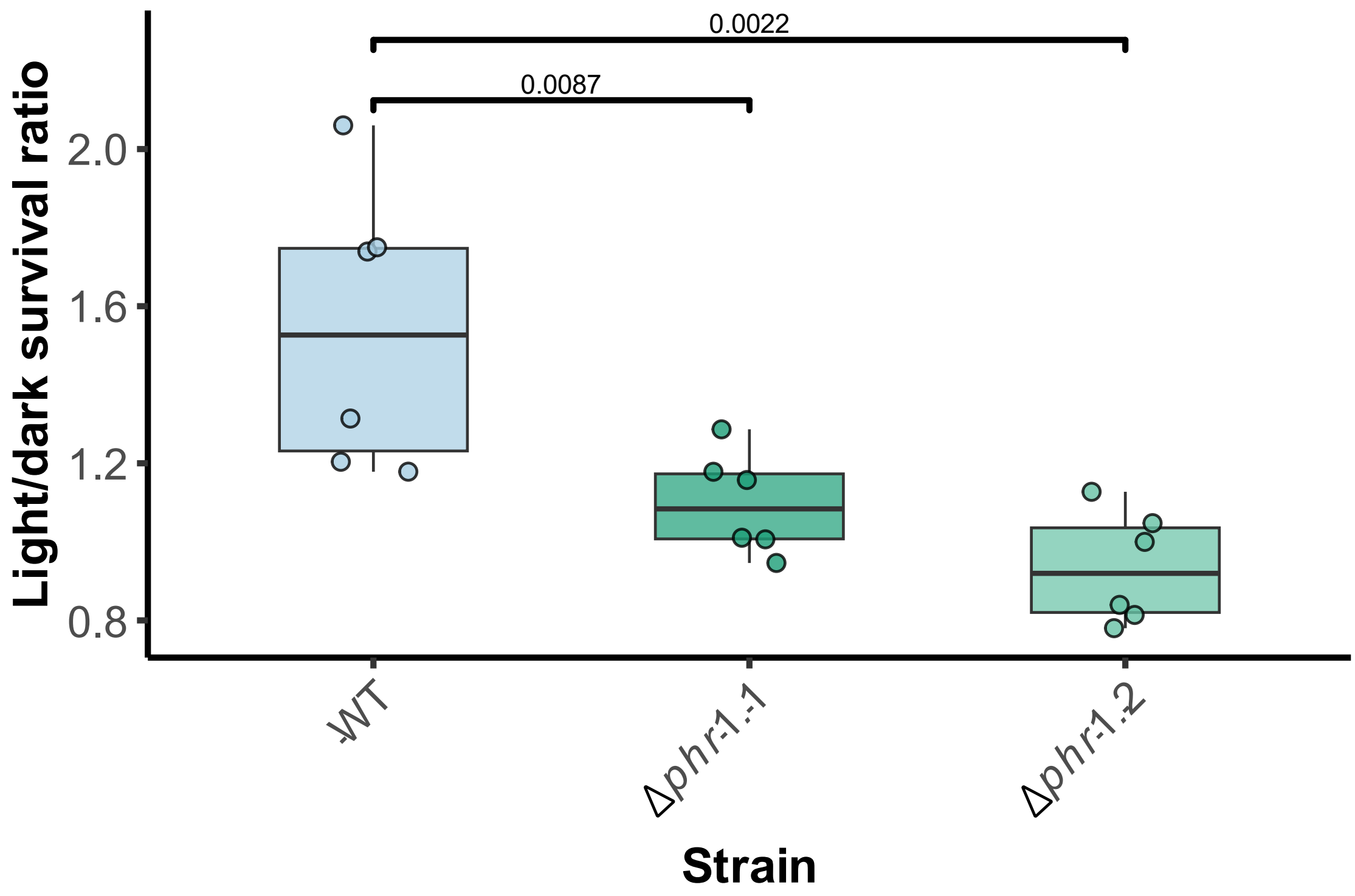

**Figure S9: Expression of *phr1* during vegetative growth and fruiting body initiation in *C. cinerea*.** Grey and yellow backgrounds indicate dark and light phases of the experiment, respectively. Expression is visualized as A) log2(count per million read) and B) raw counts. Data were obtained from the CopciDB (https://mushroomdb.brc.hu/).

**A**

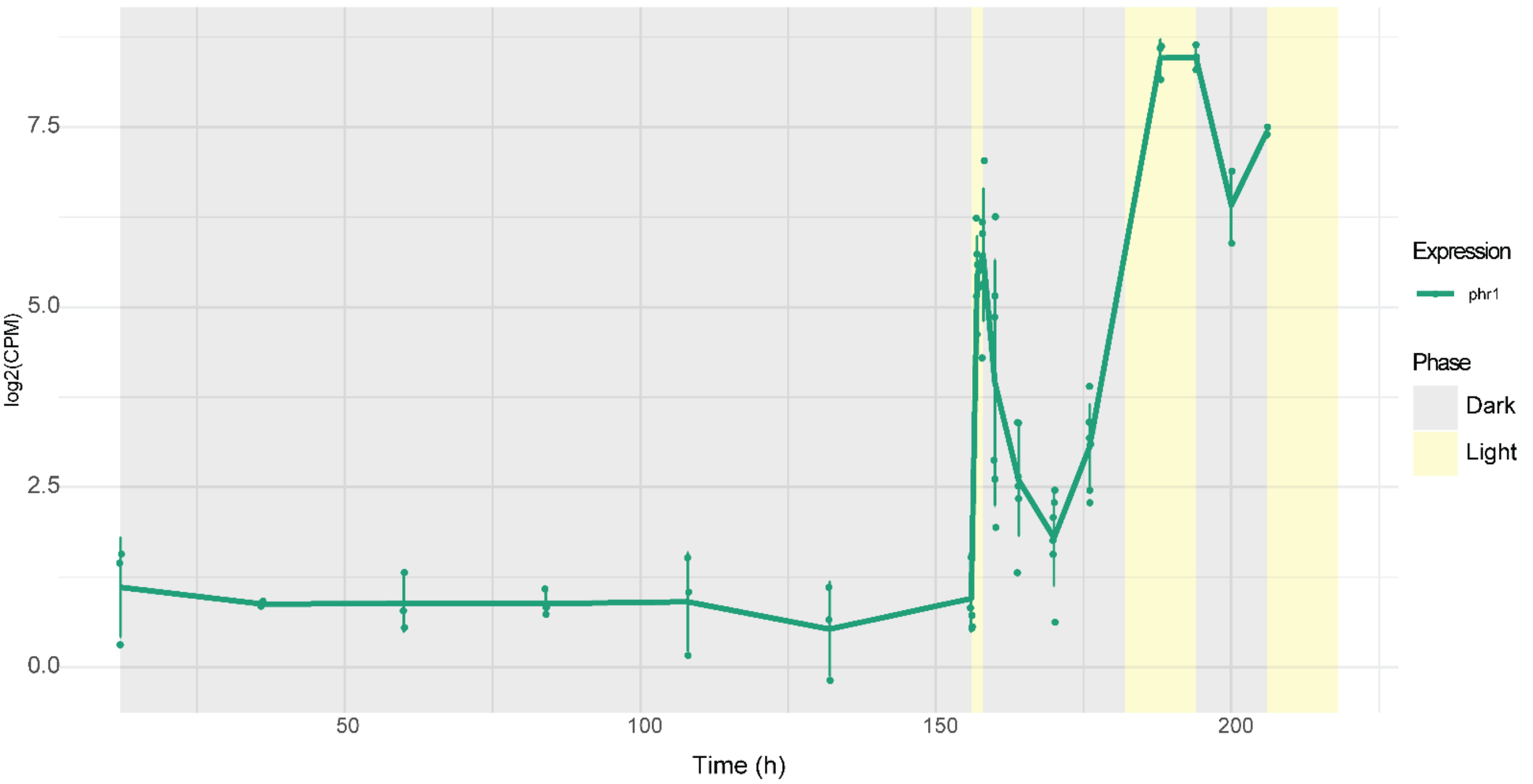


**B**

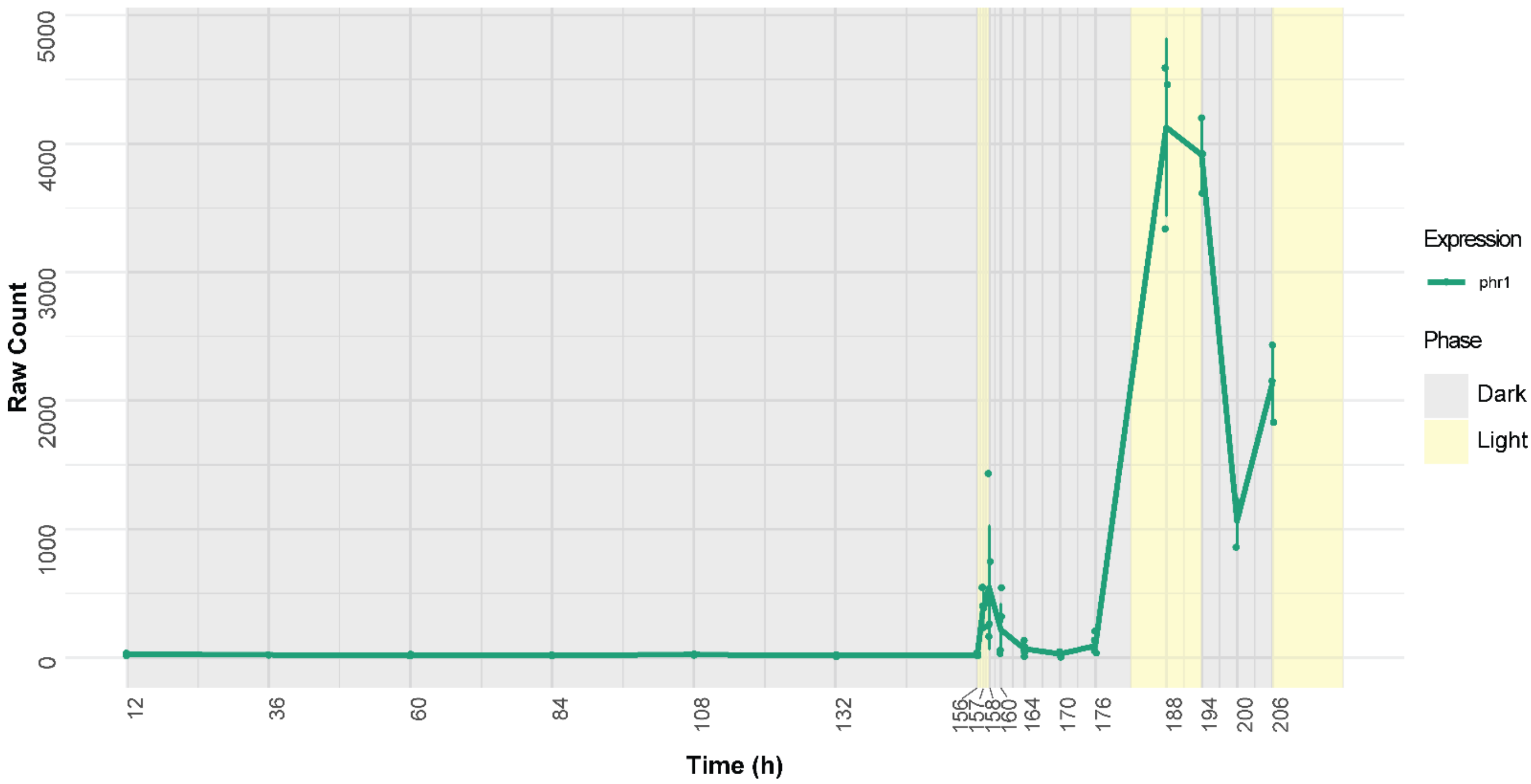

**Figure S10: Effect of Phr1 on the expression of *dst1*.** Relative expression of *dst1* (WC1) in the Δ*phr1* and in the *phr1*OE strains was determined relative to the expression of *dst1* in the WT strain under the same irradiation. The expression of *dst1* is normalized to the *dst1* level determined under the respective irradiation in the wild type. Individual dots on the IQR boxes represent the means of biological replicates (n=3-5) calculated from 2 technical replicates in each batch (k=2).

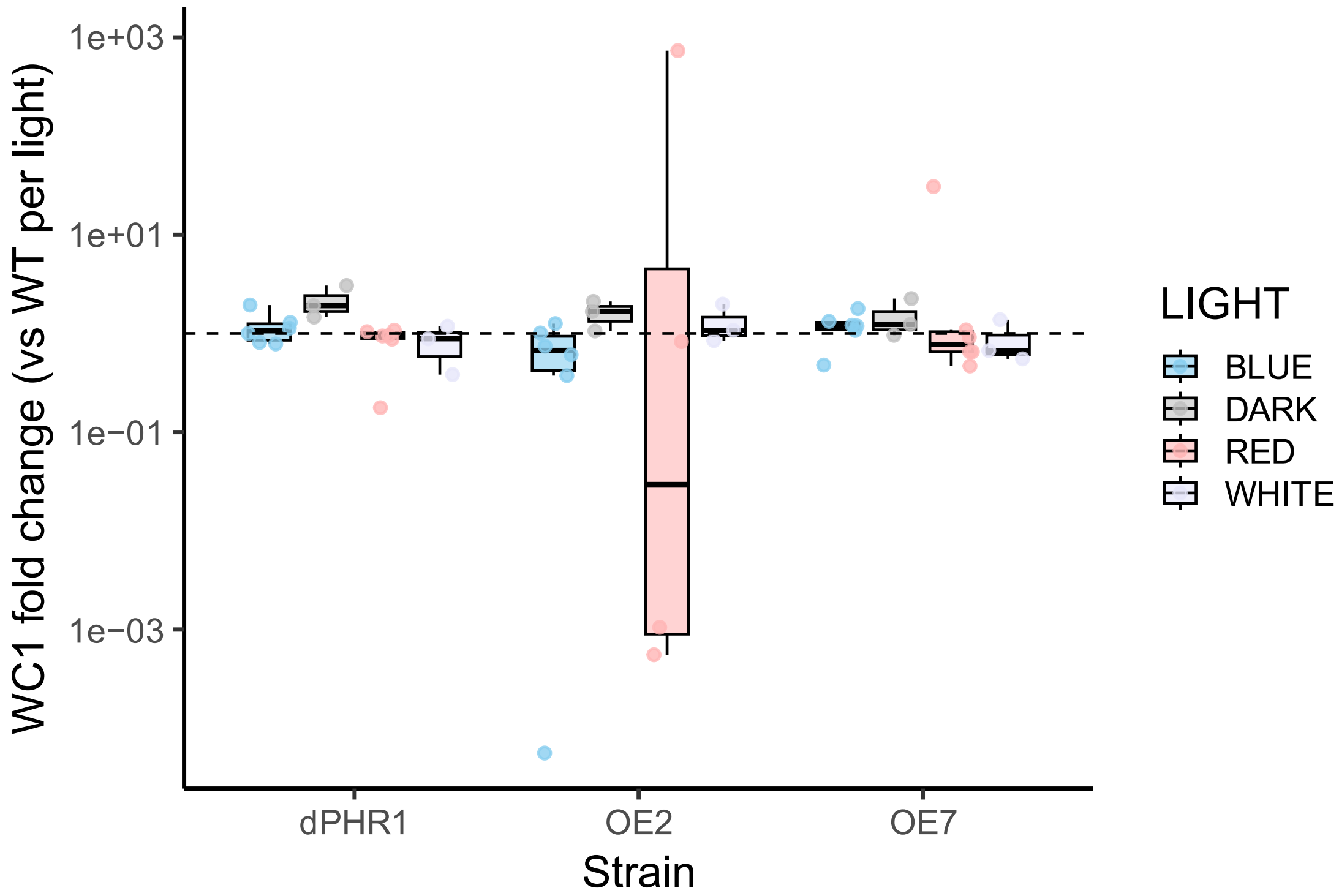

**Figure S11: Deletion and overexpression of *phr1* had no effect on the vegetative growth rate of *C. cinerea*.** Strains were grown on YMG media for 96 hours. We then calculated the growth rate (mm/h) from the radius of the colony. Bar colors represent the light irradiation during vegetative growth, in the dark, under white (25 µmol $m^{-2}$ $s^{-1}$) monochromatic red (λmax = 667 nm, 40 µmol $m^{-2}$ $s^{-1}$) and blue (λmax = 470 nm, 40 µmol $m^{-2}$ $s^{-1}$) irradiation. Error bars indicate the standard deviation of the three biological replicates (n=3), each calculated from two technical replicates (k=2).

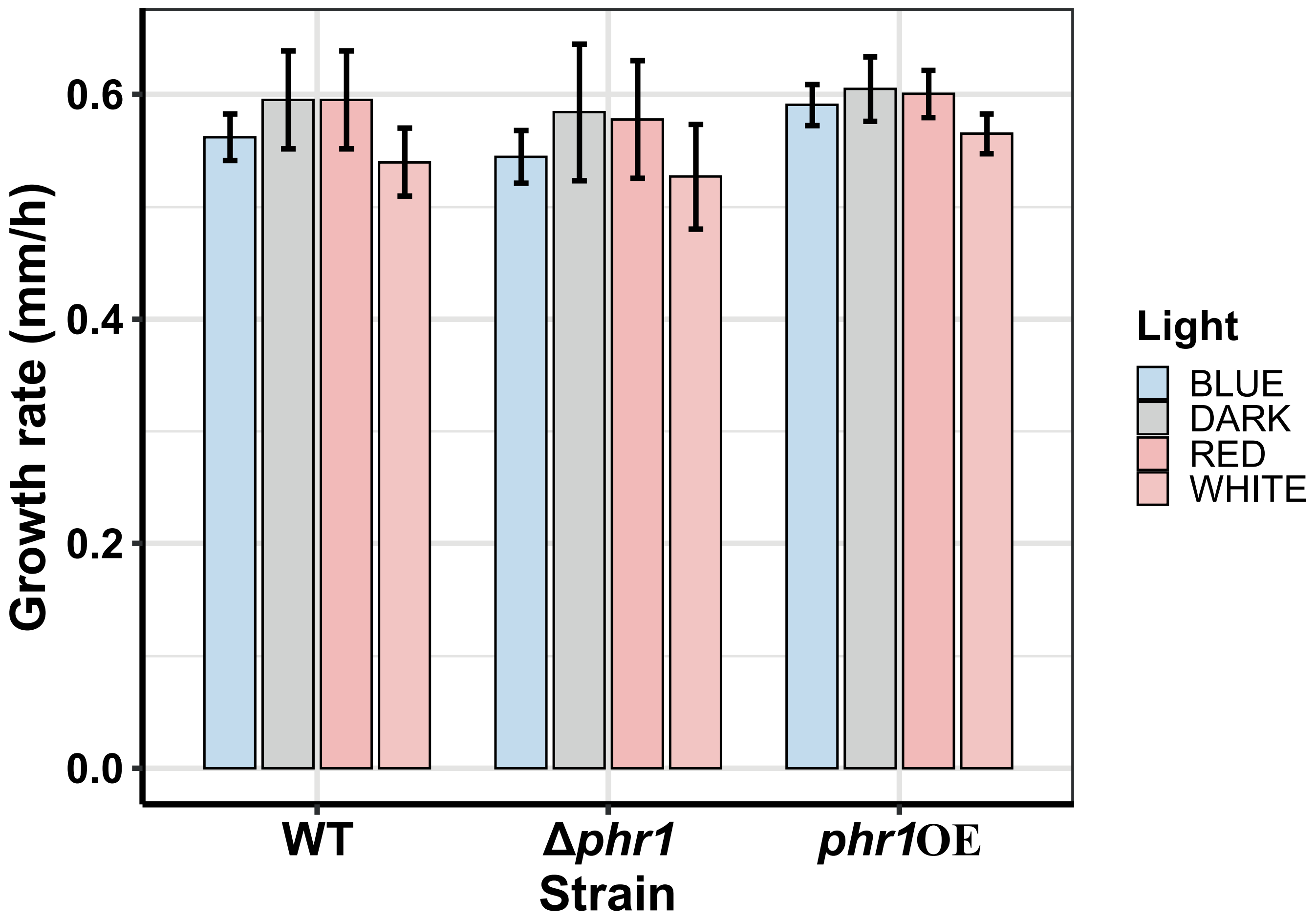

**Figure S12: Phr1 has no significant effect on oidium production.** The effect of Phr1 on oidia production under different wavelengths of light was investigated in the wt, *Δphr1*, *Δdst1*, *phr1OE* strains. The strains were grown on YMG media in the dark for 72 hours at 28°C then moved to monochromatic blue (λmax = 470 nm, 40 µmol $m^{-2}$ $s^{-1}$), monochromatic red (λmax = 667 nm, 40 µmol $m^{-2}$ $s^{-1}$) and white light (25 µmol m-² s-¹) at 28°C for 48 hours or left in the dark. Oidia were harvested and counted, and the number of oidia was determined relative to the colony diameter. Boxplots show the interquartile range (IQR); the thick horizontal line indicates the median. Each box represents three biological replicates (n = 3), each derived from two technical replicates (k = 2).

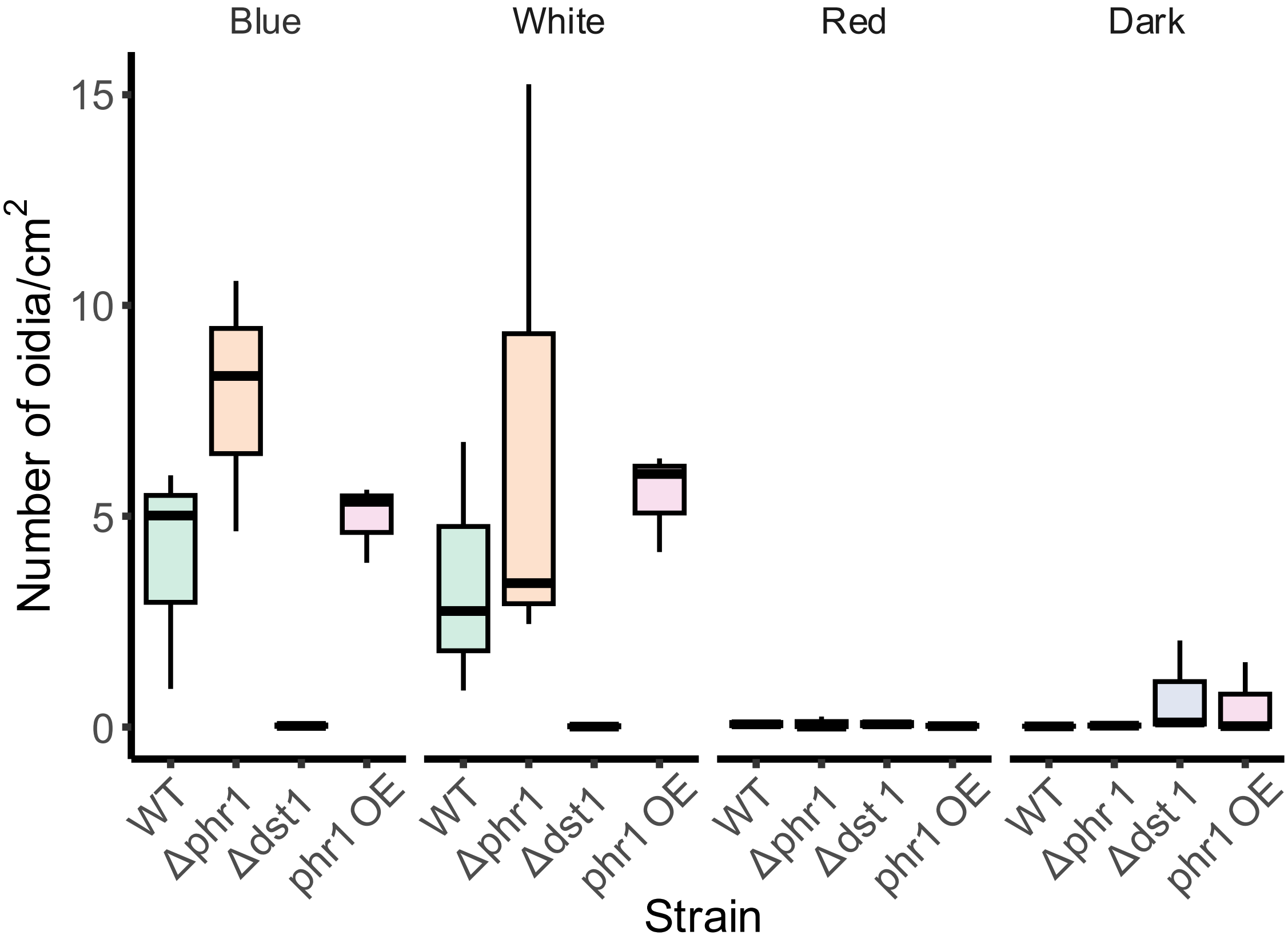

**Figure S13: Expression of *phr1* (PHR1) and *dst1* (WC1) during fruiting body formation in different Agaricomycetes species.** Protein IDs corresponding to each gene in each species are indicated in the color key. Developmental stages are abbreviated as follows: VM – vegetative mycelium, H – hyphal knot, P1/2/3 – stage 1/2/3 primordium, YFB – young fruiting body, FB/MFB mature fruiting body, D – dedifferentiated cap. Parts of the fruiting bodies are abbreviated as follows: C/K/PI– cap, L – gills, S/ST/T – stipe, V – cuticle. Details on the calculation of FPKM values and dataset sampling are provided in Nagy et al.[32]

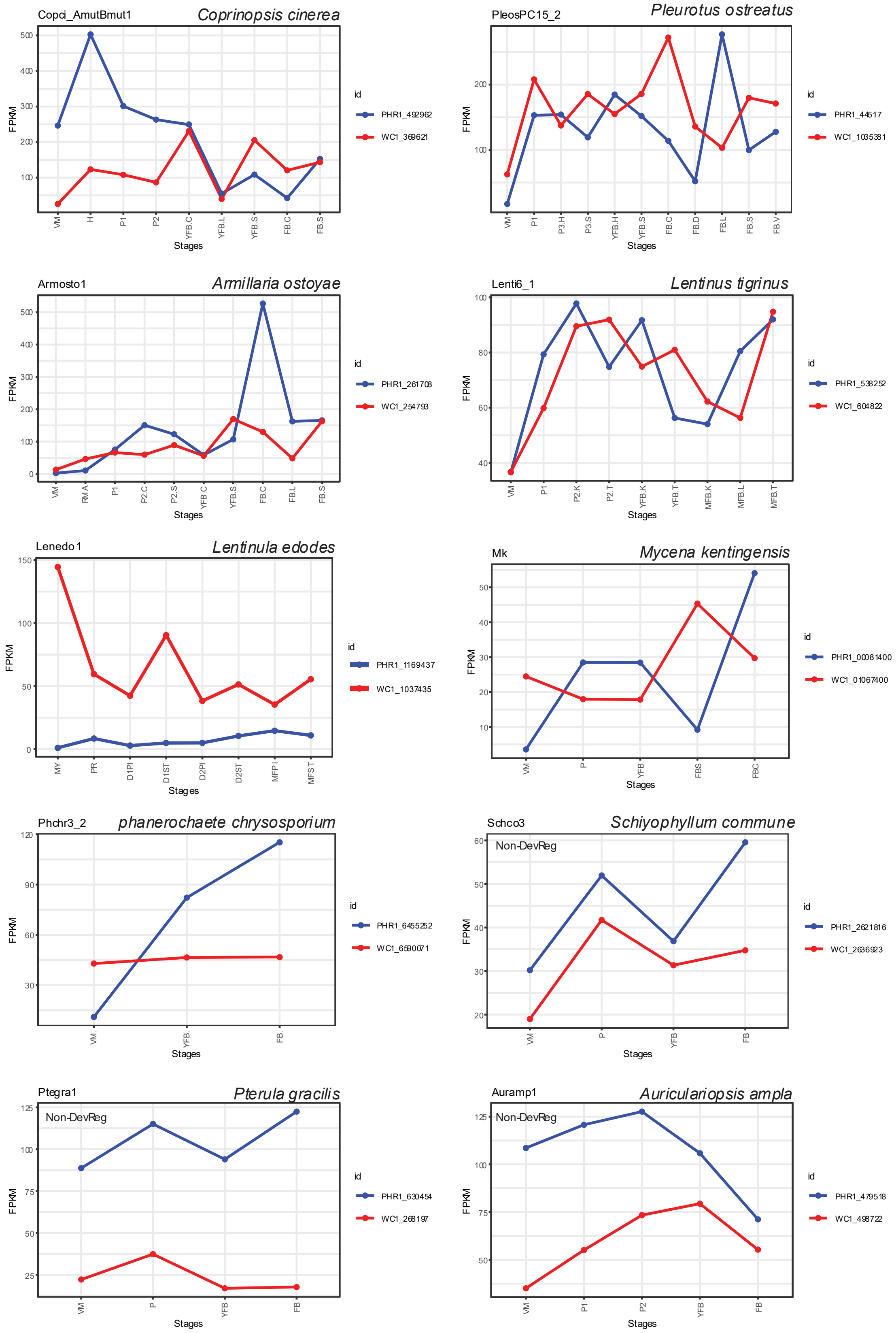

Supplement: Supplementary file 1 [file mmc1.pdf]
